# Exome sequencing data reanalysis of 200 hypertrophic cardiomyopathy patients: the HYPERGEN French cohort 5 years after the initial analysis

**DOI:** 10.3389/fmed.2024.1480947

**Published:** 2024-10-31

**Authors:** Hager Jaouadi, Victor Morel, Helene Martel, Pierre Lindenbaum, Lorcan Lamy de la Chapelle, Marine Herbane, Claire Lucas, Frédérique Magdinier, Gilbert Habib, Jean-Jacques Schott, Stéphane Zaffran, Karine Nguyen

**Affiliations:** ^1^Aix Marseille Université, INSERM, Marseille Medical Genetics (MMG), U1251, Marseille, France; ^2^Department of Medical Genetics, La Timone Hospital, AP-HM, La Timone Children’s Hospital, Marseille, France; ^3^Department of Cardiology, La Timone Hospital, AP-HM, Marseille, France; ^4^Nantes Université, CHU Nantes, CNRS, INSERM, l’institut du Thorax, Nantes, France

**Keywords:** HCM, exome reanalysis, sarcomeric genes, non-sarcomeric genes, novel-associated genes, FHOD3, TRIM63, SVIL

## Abstract

**Background:**

Approximately half of hypertrophic cardiomyopathy (HCM) patients lack a precise genetic diagnosis. The likelihood of identifying clinically relevant variants increased over time.

**Methods:**

In this study, we conducted a gene-centric reanalysis of exome data of 200 HCM cases 5 years after the initial analysis. This reanalysis prioritized genes with a matched HCM entry in the OMIM database and recently emerging HCM-associated genes gathered using a text mining-based literature review. Further classification of the identified genes and variants was performed using the Clinical Genome Resource (ClinGen) resource and American College of Medical Genetics and Genomics (ACMG) guidelines to assess the robustness of gene–disease association and the clinical actionability of the prioritized variants.

**Results:**

As expected, the majority of patients carried variants in *MYBPC3* and M*YH7* genes, 26% (*n* = 51) and 8% (*n* = 16), respectively, in accordance with the initial analysis. The vast majority of pathogenic (P) and likely pathogenic (LP) variants were found in *MYBPC3* (22 out of 40 variants) and *MYH7* (8 out of 16 variants) genes. Three genes—not included in the initial analysis—were identified: *SVIL*, *FHOD3*, and *TRIM63*. Considering only patients with unique variants in the last three genes, there was a 9% enhancement in variant identification. Importantly, *SVIL* variant carriers presented apical and septal HCM, aortopathies, and severe scoliosis for one patient. Ten patients (5%) carried variants in the *FHOD3* gene, six in hotspot regions (exons 12 and 15). We identified seven variants within the *TRIM63* gene in 12 patients (6%). Homozygous variants were detected in 2.5% of the cohort in *MYBPC3* (*n* = 1), *MYL3* (*n* = 1), and *TRIM63* (*n* = 3) genes.

**Conclusion:**

Our study revealed that no variants were found in the *ACTC1*, *TPM1*, and *TNNI3* genes in the HYPERGEN cohort. However, we identified variants in five out of the eight HCM core genes, with a high prevalence in young patients. We identified variants in three recent HCM-associated genes (*SVIL*, *FHOD3*, and *TRIM63*) in 35 patients, with 18 patients carrying unique variants (9%). Our results further emphasize the usefulness of exome data reanalysis, particularly in genotype-negative patients.

## Introduction

Hypertrophic cardiomyopathy (HCM) is an inherited cardiac disease, defined by left ventricular (LV) wall thickness greater than 15 mm, in the absence of other loading conditions that could explain the hypertrophy (1). The degree, localization, and distribution of the hypertrophy are variable (2). The LV systolic function can be preserved, increased, or reduced (2). Consequently, HCM is characterized by a phenotypic heterogeneity that could be partly explained by the heterogeneity of the genetic underlying etiology.

The estimated prevalence of HCM is 1:500 in the general population based on the recognition of the disease phenotype (3–5). However, considering familial transmission, genotype-positive cases for sarcomeric genes, and subclinical cases, a higher prevalence (1:200) is reported (6).

The clinical manifestations of HCM typically occur between the ages of 20 and 40, although they can develop at any age (7). It is noteworthy that 50–60% of cases are diagnosed after the age of 30, reflecting the variable penetrance and expressivity of the disease (3). The average life expectancy for HCM patients is typically favorable, approaching 70 years in effectively managed cases (6), although outcomes are greatly dependent on the presence of risk factors for complications such as early onset of the disease, arrhythmias, and heart failure. The annual risk of sudden cardiac death (SCD) in high-risk patients is estimated to range from 0.5 to 1%, with the highest risk observed in young cases under 30 years of age (8).

HCM is widely recognized as a sarcomeric disease since the vast majority of patients carry variants within the eight core genes encoding sarcomeric proteins (9). Indeed, the myosin-binding protein C (*MYBPC3*) and the *β*-myosin heavy chain (*MYH7*) genes account for 50–70% of HCM cases that undergo genetic testing (1–3, 10, 11). The remaining patients harbor variants in other sarcomeric genes, such as myosin light chain (MLC) genes (*MYL2* and *MYL3*), troponin encoding genes (*TNNT2* and *TNNI3*), tropomyosin 1 (*TPM1*), and actin *α*-cardiac muscle 1 (*ACTC1*) (4, 11, 12). Collectively, actionable variants in sarcomeric-positive (sarc+) patients account for >90% of the totality of pathogenic (P) variants in HCM patients (12). Of note, these percentages vary widely depending on the studied populations, the clinical profiling, the sequencing method used (panel, whole-exome sequencing [WES], etc.), and variant prioritization criteria.

Several additional genes have been linked to HCM encoding non-sarcomeric proteins, including, but not restricted to, proteins of the Z-disk (*ACTN2*, *TCAP*, and *VCL*), calcium handling genes (*TNNC1*, *RYR2*, *JPH2*, *PLN*), and the proteasome (*TRIM63*) (4). Similarly to the sarcomeric genes, both inheritance models are reported in these genes with a predominance of the autosomal dominant pattern (12, 13). However, the contribution of these genes is minor, ranging from 0.06 to 8.7% based on combined published data involving genes with strong, moderate, and weak evidence of causality and HCM-associated genes supported only by functional data (13). These findings reinforce the fact that sarcomeric genes predominantly cause HCM and emphasize the significant proportion of the missing heritability in HCM (13). Therefore, up to 50% of HCM patients do not have an identifiable pathogenic disease-causing variant (14, 15). So far, this missing heritability is increasingly explained by the fact that sarcomeric-negative (Sarc-) and genotype-negative patients have a non-Mendelian or near-Mendelian disease caused by a joint effect of genetic and non-genetic factors (13, 16). Thus, the additive effect of allelic heterogeneity and additional genetic variants, along with variants in cis causing allelic imbalance, could also partially explain the missing heritability, the phenotypic variability, and the incomplete penetrance. In this line, many studies have suggested the oligogenic inheritance model in HCM patients lacking an identifiable highly penetrant variant (16–20). Furthermore, rare variants are broadly acknowledged among the plausible sources of missing heritability and the likely contributors to the oligogenic inheritance rather than common variants. Indeed, variants with strong effects are expected to be kept under selective pressure and remain at low to extremely low frequencies in the population (21, 22). Altogether, incomplete penetrance, variable expressivity, and missing heritability in HCM make the genetic diagnosis and clinical prognosis challenging.

The efficiency of WES reanalysis has been demonstrated to improve genetic testing yield, especially by adding the newly associated genes previously unrecognized as clinically relevant. It is estimated that the genetic diagnostic yield could be enhanced by approximately 15% when including new disease-gene associations, up-to-date software, variant frequency databases, and text mining for genetic and clinical re-evaluation (23). Moreover, it is recommended that the dataset initially analyzed over 2 years ago should be prioritized for reanalysis (24).

In this study, we sought to refine the initial Hypertension Genetic Epidemiology Network (HYPERGEN) analysis by focusing on HCM-causing genes (that are known and recently associated). Additionally, we assessed the usefulness of our reanalysis regarding the clinical actionability of the identified variants by applying the American College of Medical Genetics and Genomics/Association of Molecular Pathologists (ACMG/AMP) criteria.

## Methods

This study was approved by an institutional review committee. All subjects gave informed consent for genetic studies. WES was performed on the NGS Illumina HiSeq2500 platform using Agilent SureSelect V6 technology (Genwiz, United States) (25).

### The HYPERGEN cohort

The HYPERGEN cohort included 132 men and 68 women (mean age, 55 years; range, 19–91 years; 86.5% above 40; 34% familial cases). The patients were enrolled in five French centers: Marseille, Bordeaux, Paris, Dijon, and Rennes.

A definite echocardiographic evidence of HCM was considered based on the measurements of maximal wall thickness ≥ 15 mm in sporadic and > 13 mm in familial cases without dilatation or any cardiovascular comorbidities or systemic disease.

None of the patients was reported by the clinicians as syndromic or likely syndromic. However, the first HYPERGEN study identified one novel variant in the *GLA* gene (p.Leu311Profs*4), and the clinical re-evaluation of the patient confirmed a Fabry disease diagnosis. A second case with the disease-causing variant *TTR*: p.Val50Met has been reported with transthyretin cardiac amyloidosis (25). Of note, this study’s variant prioritization strategy is different from the first HYPERGEN analysis. Thus, an important caveat to our reanalysis is that an accurate comparison of the genetic diagnosis yield cannot be assessed as the initial analysis pipeline differs from this reanalysis. Additionally, since we do not have access to the variant coordinates identified in the first analysis, it is impossible to determine whether any variants have been downgraded or upgraded. The significant differences are detailed in the following.

### Initial HYPERGEN analysis vs. the 5 years interval analysis

In the initial HYPERGEN study, bioinformatic analyses were performed by retaining exonic and intronic variants with a minor allele frequency (MAF) lower than 1% and predicted as P or likely pathogenic LP mainly according to the Universal Mutation Database (UMD) predictor. The first step of WES data analysis consisted of searching for variants in a virtual panel of 167 genes involved in cardiomyopathies and other various cardiac hereditary diseases (25).

**Figure 1:**
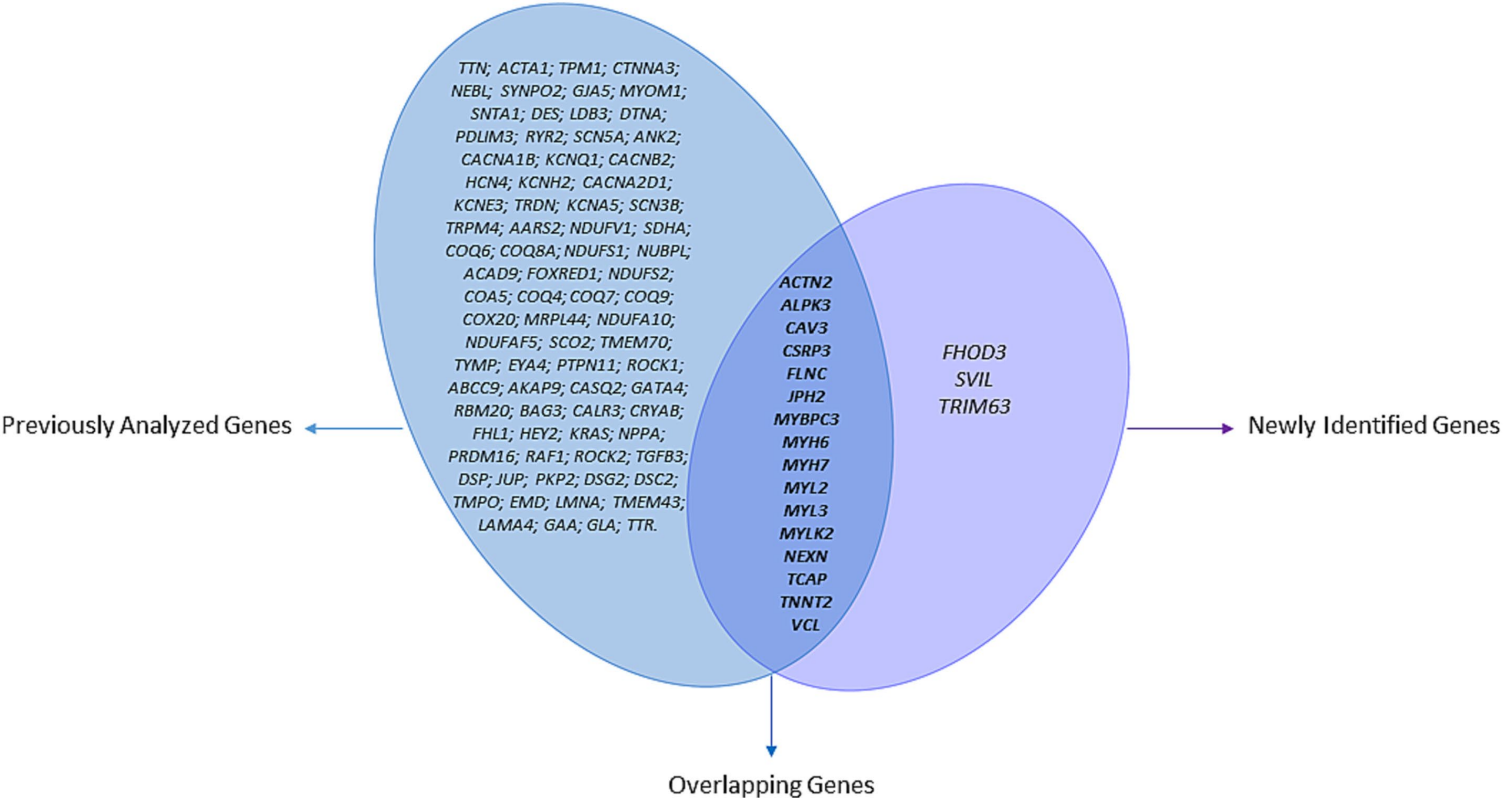
Venn diagram visualization of genes across initial and 5-year analyses. The “previously analyzed genes” section includes genes from the 167-gene panel used in the original HYPERGEN study; the “overlapping genes” section contains genes identified in both the initial and current analyses; the “newly identified genes” section consists of genes associated with HCM between 2019 and 2024.

In contrast, in this reanalysis, priority was given to genes with a matched HCM entry in Mendelian disease in the Online Mendelian Inheritance in Man (OMIM) database and the ClinGen^1^ as well as recently emerging HCM-associated genes gathered using text mining-based literature review from 2016 to 2023 (Figure 1).

More importantly, variant pathogenicity was assessed according to ACMG/AMP guidelines, and the consistency of a predicted deleterious effect by at least 3 prediction tools and a high combined annotation-dependent depletion (CADD) score.

As aforementioned, the initial HYPERGEN analysis showed two HCM phenocopies. In this study, a preanalysis included the significant syndromic genes and genes yielding known HCM phenocopies. None of the variants identified within these genes were classified as P or LP according to ACMG or reported in ClinVar database (data not shown). Moreover, all these variants were found along with other HCM-relevant variants. Thus, in this reanalysis, variants in genes causing syndromic HCM and phenocopies are not included. The *TTN* gene is not included as well.

The Venn diagram shown in Figure 1 illustrates the relationship between the genes previously prioritized in the first analysis and those reanalyzed in this study. The overlapping section represents genes shared between both analyses.

### Variant filtering, prioritization, and sanger validation

The first criterion of this reanalysis is variant rarity. Thus, a filtering allele frequency of <1% in gnomAD database was applied. Subsequently, intergenic, intronic, and synonymous variants were removed. The remaining variants were prioritized based on their *in silico* predicted impact on protein function. This analysis’s primary scoring tool is the CADD (26). Indeed, variants predicted as damaging or probably damaging, deleterious, disease-causing by PolyPhen-2 and SIFT, and/or MutationTaster while also having a CADD score > 15 were considered as variants of high confidence of pathogenicity (27–29). Of note, up to two-thirds of the variants of uncertain significance (VUS) in confirmed sarcomeric genes are considered causal of HCM (30). Thus, suspicious VUS with some evidence of causality (a rarity, VUS within well-established HCM genes, mutational hotspots…) is reported in this reanalysis.

Finally, to gauge the clinical actionability of the detected variants, a subsequent classification according to ACMG criteria was performed (31).

All the prioritized variants within the core and minor HCM genes were validated by Sanger sequencing when DNA samples were available (Supplementary File 1).

### Biological pathway and gene ontology analyses

Given a large number of variants, even after the filtering steps, variants within HCM-minor genes could be missed. To facilitate and ensure the identification of these variants, a biological pathway analysis including Reactome and KEGG databases as well as gene (GO) and human phenotype (HPO) ontologies terms was carried out using VarAFT software (32–36). For example, querying the HPO and GO databases with the keywords “cardiomyopathy phenotype” and “cardiac hypertrophy” results in 3 HPO term identifiers (HP:0001639, HP:0005157, HP:0031992), 27 disease terms (matching all the OMIM phenotypes) and 83 additional genes. VarAFT software allowed us to query each variant call format (VCF) file for all the possible combinations.

### French control cohort

French healthy controls (*n* = 960) were queried to compare variants allele frequencies. Exons coordinate on GRCh37 were extracted for the genes of interest and extended by 50 bases. Those regions were called using GATK (4.3.0.0) with a set of Binary Alignment Map (BAM) files using a nextflow-based pipeline for joint-genotyping.^2^ Resulting VCF was annotated with snpEff (37) and jvarkit/GnomAD.^3^ Variants known to be filtered in gnomAD or having an allele frequency greater than 1% in the non-finish population were excluded. Protein truncating and splice site variants were kept using jvarkit/vcffilterso.

## Results

### Variant identification among the definitive HCM genes

#### *MYBPC3* and *MYH7* genes: the permanently significant HCM genes

Definitive HCM genes with a well-established disease association are mainly the eight sarcomeric genes with strong evidence of causality (*MYBPC3*, *MYH7*, *TNNT2*, *TNNI3*, *TPM1*, *ACTC1*, *MYL3*, and *MYL2*) (38, 39). In our analysis, variants were found in five out of these eight well-established genes.

**Figure 2:**
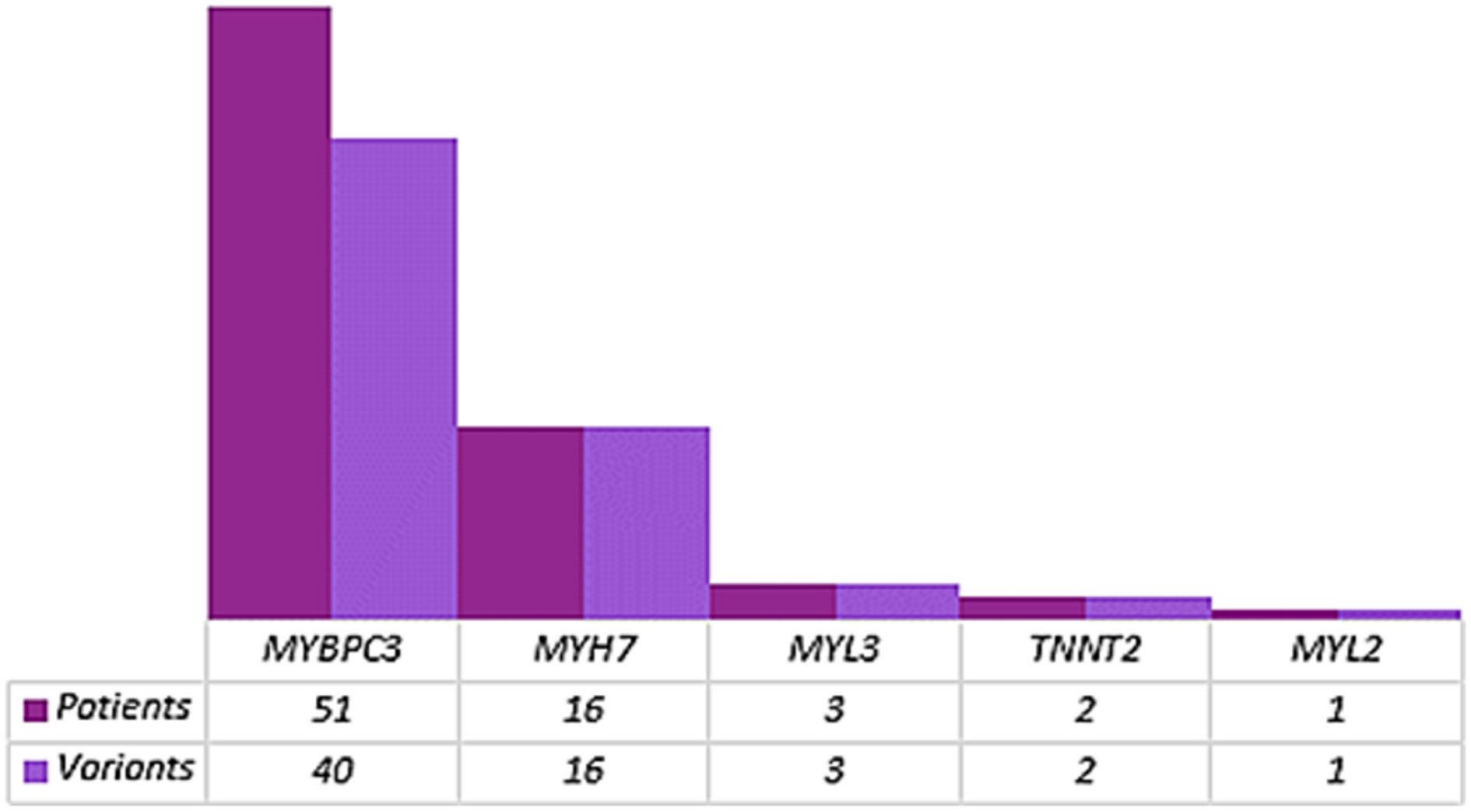
Variant distribution within HCM well-established genes. The number of patients harboring variants in each HCM core gene is dark purple. The number of variants within each gene is indicated in light purple.

Not surprisingly, the most prevalent genes in our case cohort are *MYBPC3* and *MYH7* genes, which is in accordance with the initial HYPERGEN study (Figure 2).

Variants coordinates are detailed in Tables 1–4.

**Table 1:** Variants identified in the *MYBPC3* gene (transcript NM_000256, MANE select).

Patient ID	Genotype	Variant type	AA change	rs ID	CADD	ACMG/ClinVar	GnomAD-AF	Additional variant
HCM-1	Het	Splicing	Ex4:c.505 + 2 T > A	NA	23.5	LP/NA	NA	Unique variant
HCM-2	Het	Stopgain	Ex4:p.(Ser139*)	rs730880704	35	LP/LP	NA	Unique variant
HCM-3	Het	Missense	Ex5:p.(Arg177His)	rs201012766	23.5	B/B	0.001201	Unique variant
HCM-4	Het	Stopgain	Ex5:p.(Gln205*)	rs397516061	36	LP/P	NA	Unique variant
HCM-5	Het	Missense	Ex5:p.(Leu183Ile)	NA	24.3	VUS/NA	NA	*MYBPC3*:c.1928-2A > G
HCM-6	Het	Missense	Ex5:p.(Ser217Gly)	rs138753870	14.62	B/Conflicting	0.001693	*MYBPC3*:p.(Arg943*)
HCM-7	Het	Missense	Ex5:p.(Val189Ile)	rs11570052	20.0	B/B	0.002459	Unique variant
HCM-8	Het	Missense	Ex5:p.(Val189Ile)	rs11570052	20.0	B/B	0.002459	Unique variant
HCM-9	Het	Missense	Ex6:p.(Arg238Cys)	rs771143409	33	VUS/VUS	0.00001615	*ACTN2*: p.(Thr347Met)
HCM-10	Het	Missense	Ex6:p.(Glu258Lys)	rs397516074	28.8	P/P	0.00002199	Unique variant
HCM-11	Het	Missense	Ex8:p.(Arg281Gly)	rs371711564	23.4	VUS/VUS	NA	Unique variant
HCM-12	Het	Frameshift deletion	Ex11:p.(Phe305ProfsTer27)	rs397516080	NA	LP/P	NA	Unique variant
HCM-13	Het	Missense	Ex12:p.(Arg326Gln)	rs34580776	24.0	B/B	0.004359	Unique variant
HCM-14	Het	Missense	Ex12:p.(Gly341Ser)	rs397515881	34	VUS/VUS	0.00004999	*MYBPC3*: p.(Pro955ArgfsTer95)
HCM-15	Het	Missense	Ex12:p.(Val321Met)	rs200119454	26.5	VUS/Conflicting	0.0003297	Unique variant
HCM-16	Het	Missense	Ex13:p.(Arg382Trp)	rs11570076	34	B/B	0.004239	Unique variant
HCM-17	Het	Splicing	Ex14:c.1351 + 2 T > C	rs397515897	23.1	LP/P	NA	Unique variant
HCM-18	Het	Frameshift deletion	Ex14:p.(Val437GlyfsTer13)	rs397515896	NA	LP/P	NA	Unique variant
HCM-19	Het	Splicing	Ex16:c.1624 + 4A > T	rs397515916	NA	LP/P	0.00001334	Unique variant
HCM-20	Het	Missense	Ex16:p.(Arg495Gln)	rs200411226	30	LP/P	0.00002408	Unique variant
HCM-21	Het	Missense	Ex16:p.(Arg495Gln)	rs200411226	30	LP/P	0.00002408	Unique variant
HCM-22	Het	Missense	Ex16:p.(Arg495Gly)	rs397515905	25.4	P/P	0.000004013	Unique variant
HCM-23	Het	Missense	Ex16:p.(Arg502Trp)	rs375882485	34	P/P	0.00004632	Unique variant
HCM-24	Het	Missense	Ex16:p.(Arg502Trp)	rs375882485	34	P/P	0.00004632	Unique variant
HCM-25	Het	Missense	Ex16:p.(Arg502Trp)	rs375882485	34	P/P	0.00004632	Unique variant
HCM-26	Hom	Missense	Ex17:p.(Ala562Thr)	rs397515919	31	VUS/VUS	NA	Unique variant
HCM-27	Het	Missense	Ex17:p.(Arg597Gln)	rs727503195	35	P/P	0.00002996	*FHOD3*: p.(Arg637Gln)
HCM-28	Het	Missense	Ex18:p.(Glu611Lys)	rs730880555	35	VUS/Conflicting	0.00002350	Unique variant
HCM-29	Het	Splicing	Ex20:c.1928-2A > G	rs397515937	23.8	P/P	NA	Unique variant
HCM-30	Het	Splicing	Ex20:c.1928-2A > G	rs397515937	23.8	P/P	NA	Unique variant
HCM-31	Het	Splicing	Ex20:c.1928-2A > G	rs397515937	23.8	P/P	NA	Unique variant
HCM-32	Het	Splicing	Ex20:c.1928-2A > G	rs397515937	23.8	P/P	NA	Unique variant
HCM-5	Het	Splicing	Ex20:c.1928-2A > G	rs397515937	23.8	P/P	NA	*MYBPC3:* p.(Leu183Ile)
HCM-33	Het	Splicing	Ex20:c.1928-2A > G	rs397515937	23.8	P/P	NA	Unique variant
HCM-34	Het	Splicing	Ex20:c.1928-2A > G	rs397515937	23.8	P/P	NA	Unique variant
HCM-35	Het	Missense	Ex22:p.(Asp770Asn)	rs36211723	34	LP/Conflicting	0.00001606	Unique variant
HCM-36	Het	Frameshift insertion	Ex22:p.(Lys754GlufsTer79)	rs774521272	NA	LP/P	0.000008025	Unique variant
HCM-37	Het	Frameshift insertion	Ex22:p.(Lys754GlufsTer79)	rs774521272	NA	LP/P	0.000008025	Unique variant
HCM-38	Het	Frameshift insertion	Ex23:p.(Trp792ValfsTer41)	rs397515963	NA	P/P	NA	Unique variant
HCM-39	Het	Frameshift insertion	Ex23:p.(Trp792ValfsTer41)	rs397515963	NA	P/P	NA	*SVIL*: p.(Ala1496Asp)
HCM-40	Het	Missense	Ex24:p.(Ala833Val)	rs3729952	26.9	B/B	0.002538	*MYH6*: p.(Arg1532Leu) *SVIL*: p.(Thr1630Ser)
HCM-41	Het	Missense	Ex24:p.(Arg810His)	rs375675796	35	LP/Conflicting	0.00004816	Unique variant
HCM-6	Het	Stopgain	Ex26:p.(Arg943*)	rs387907267	43	P/P	0.00001214	*MYBPC3:* p.(Ser217Gly)
HCM-42	Het	Stopgain	Ex26:p.(Gln969*)	rs397515992	38	LP/P	NA	Unique variant
HCM-43	Het	frameshift deletion	Ex26:p.(Pro955ArgfsTer95)	rs397515990	NA	P/P	0.00003187	Unique variant
HCM-14	Het	frameshift deletion	Ex26:p.(Pro955ArgfsTer95)	rs397515990	NA	P/P	0.00003187	*MYBPC3:* p.(Gly341Ser)
HCM-44	Het	Missense	Ex28:p.(Arg1022Pro)	rs397516000	34	VUS/Conflicting	0.00002517	*MYH7*: p.(His1185Gln) *TRIM63*: p.(Cys23Tyr)
HCM-45	Het	Stopgain	Ex28:p.(Gln1061*)	rs397516005	39	LP/P	0.00001477	Unique variant
HCM-46	Het	Missense	Ex30:p.(Arg1138His)	rs187705120	35	VUS/B	0.001139	Unique variant
HCM-47	Het	Missense	Ex30:p.(Ile1131Thr)	rs370890951	22.5	B/Conflicting	0.0008403	Unique variant
HCM-48	Het	Frameshift deletion	Ex31:p.(Cys1202Leufs*35)	NA	NA	NA	NA	*FHOD3*: p.(Arg637Gln)
HCM-49	Het	stopgain	Ex32:p.(Cys1244*)	rs730880600	38	LP/P	NA	Unique variant
HCM-50	Het	Stopgain	Ex32:p.(Cys1244*)	rs730880600	38	LP/P	NA	Unique variant
HCM-51	Het	Missense	Ex32:p.(Ser1213Pro)	NA	25.3	VUS/NA	NA	Unique variant

AA, amino acid; ACMG, American College of Medical Genetics and Genomics; Het, heterozygous; Hom, homozygous; B, benign; P, pathogenic; LP, likely pathogenic; VUS, variant of uncertain significance; NA, no data available; Conflicting, conflicting interpretations of pathogenicity; CADD, combined annotation-dependent depletion scores according to CADD model v1.3; GnomAD-AF, allele frequency according to gnomAD V2.1.

**Table 2:** Variants identified in the *MYH7* gene (transcript NM_000257, MANE select).

Patient ID	Genotype	Variant type	AA change	rs ID	CADD	ACMG/ClinVar	GnomAD-AF	Additional variant
HCM-52	Het	Missense	Ex8:p.(Asn232Ser)	NA	24.8	VUS/NA	NA	*MYH6*: p.(His1526Arg) *CAV3*: p.(Thr78Met)
HCM-53	Het	Missense	Ex9:p.(Ile250Phe)	rs397516268	25.4	VUS/Conflicting	NA	Unique variant
HCM-54	Het	Missense	Ex14:p.(Arg453Cys)	rs121913625	33	P/P	NA	Unique variant
HCM-55	Het	Missense	Ex18:p.(Arg663His)	rs371898076	27.8	P/P	0.00001414	Unique variant
HCM-56	Het	Missense	Ex19:p.(Arg694Cys)	rs727504240	34	VUS/Conflicting	0.00001989	Unique variant
HCM-57	Het	Missense	Ex19:p.(Arg719Gln)	rs121913641	34	P/P	NA	*SVIL*: p.(Val1438Met)
HCM-58	Het	Missense	Ex20:p.(Gly741Arg)	rs121913632	33	P/P	0.00003185	Unique variant
HCM-59	Het	Missense	Ex20:p.(Ile736Thr)	rs727503261	24.7	P/P	NA	Unique variant
HCM-60	Het	Missense	Ex21:p.(Ala797Thr)	rs3218716	20.5	P/P	0.00002386	Unique variant
HCM-61	Het	Missense	Ex21:p.(Arg787Cys)	rs145677314	22.7	VUS/Conflicting	0.00006364	*VCL*: p.(His636Arg) *MYPN*: p.(Asp1208Gly)
HCM-62	Het	Missense	Ex21:p.(Gly768Arg)	rs727503260	35	P/P	NA	*SVIL*: p.(Ser1414Thr)
HCM-63	Het	Missense	Ex22:p.(Arg869His)	rs202141173	34	VUS/LP	0.00002386	*MYLK2*: p.(Ser510Leu) *TRIM63*: p.(Cys75Tyr)
HCM-44	Het	Missense	Ex27:p.(His1185Gln)	NA	24.0	VUS/NA	NA	*MYBPC3*:p.(Arg1022Pro) *TRIM63*: p.(Cys23Tyr)
HCM-64	Het	Missense	Ex33:p.(Glu1546Gly)	NA	29.3	VUS/NA	NA	Unique variant
HCM-65	Het	Missense	Ex35:p.(Arg1712Gln)	rs193922390	28.3	LP/P	0.00002125	Unique variant
HCM-66	Het	Missense	Ex37:p.(Glu1829Lys)	rs143562243	27.9	VUS/VUS	0.00001594	*FLNC*: p.(Arg1719Cys)

AA, amino acid; ACMG, American College of Medical Genetics and Genomics; Het, heterozygous; P, pathogenic; LP, likely pathogenic; VUS, variant of uncertain significance; NA, no data available; Conflicting: Conflicting interpretations of pathogenicity; CADD, combined annotation-dependent depletion scores according to CADD model v1.3; GnomAD-AF: allele frequency according to gnomAD V2.1.1.

**Table 3:** Variants identified in the *TNNT2* gene (transcript NM_001276345, MANE select).

Patient ID	Genotype	Variant type	AA change	rs ID	CADD	ACMG/ClinVar	GnomAD-AF	Additional variant
HCM-73	Het	Missense	Ex9:p.(Val95Met)	NA	30	VUS/LP	NA	Unique variant
HCM-74	Het	Missense	Ex16:p.(Lys283Glu)	rs1553279294	28.4	VUS/P	NA	Unique variant

AA, amino acid; ACMG, American College of Medical Genetics and Genomics; Het, heterozygous; P, pathogenic; LP, likely pathogenic; VUS, variant of uncertain significance; NA, no data available; CADD, combined annotation-dependent depletion scores according to CADD model v1.3; GnomAD-AF, Aalele frequency according to gnomAD V2.1.1.

**Table 4:** Variants identified in myosin light chain (MLC) genes.

Patient ID	Gene	Transcript	Genotype	Variant type	AA change	rs ID	CADD	ACMG/ClinVar	GnomAD-AF	Additional variant
HCM-80	*MYL2*	NM_000432	Het	Missense	Ex5:p.(Gly92Ala)	NA	25.2	VUS/NA	NA	Unique variant
HCM-77	*MYL3*	NM_000258	Hom	Missense	Ex3:p.(Ala57Asp)	rs139794067	29.8	VUS/Conflicting	0.0001627	Unique variant
HCM-78	*MYL3*	NM_000258	Het	Missense	Ex3:p.(Arg94Cys)	rs730880961	33	VUS/VUS	0.00002388	Unique variant
HCM-79	*MYL3*	NM_000258	Het	Missense	Ex5:p.(Arg163Thr)	rs752165383	25.0	VUS/VUS	0.000003998	Unique variant

AA, amino acid; ACMG, American College of Medical Genetics and Genomics; Het, heterozygous; Hom, homozygous; VUS, variant of uncertain significance; NA, no data available; Conflicting, conflicting interpretations of pathogenicity; CADD, combined annotation-dependent depletion scores according to CADD model v1.3; GnomAD-AF, allele frequency according to gnomAD V2.1.

**Figure 3:**
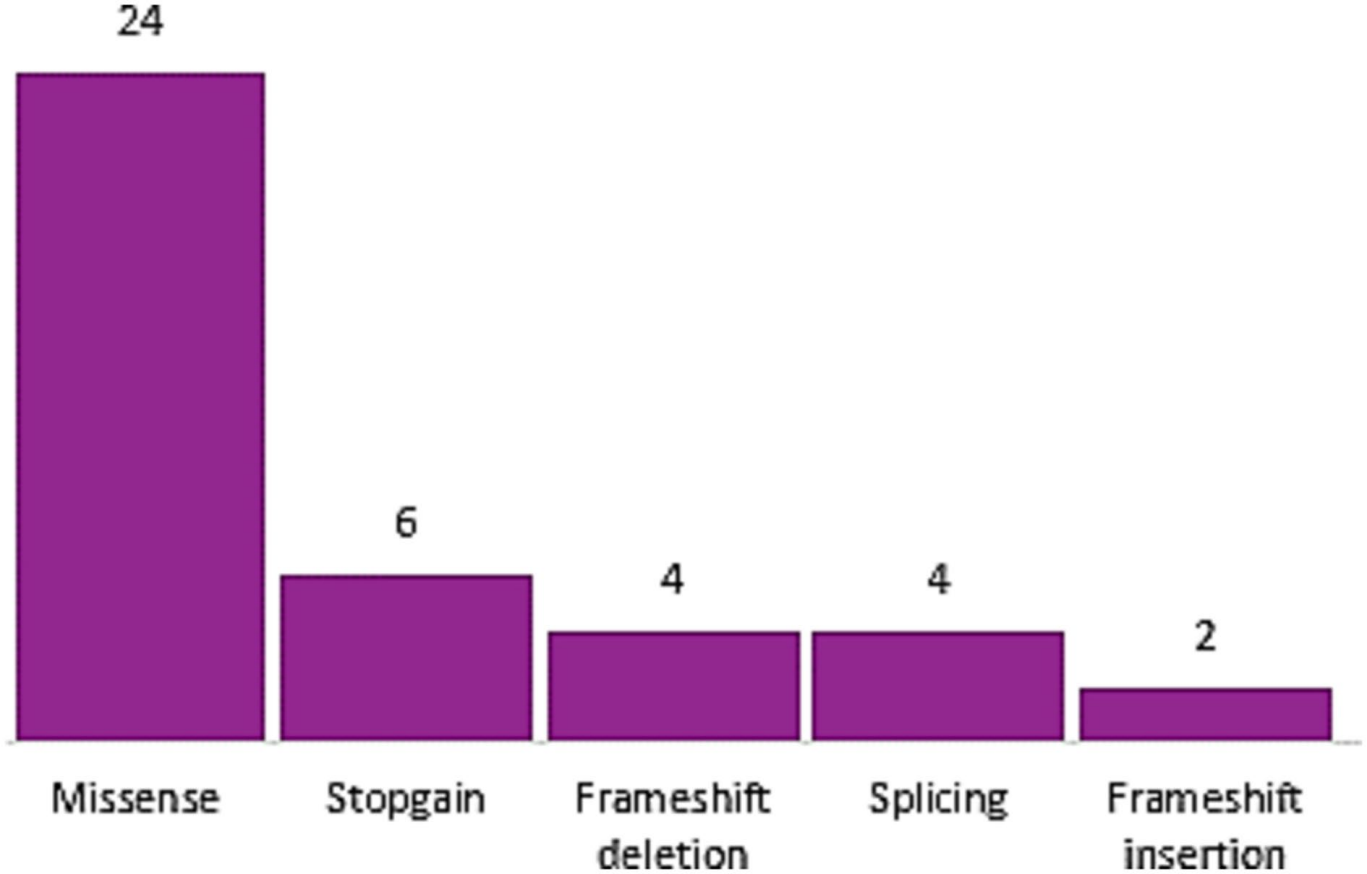
Variant type distribution of *MYBPC3* gene.

We identified 24 missense variants and 16 protein-truncating variants (PTV) in *MYBPC3* gene (Figure 2). The vast majority of the identified *MYBPC3* variants are unique (42 out of 51 patients; 82%), 3 patients are double heterozygous (HCM-5, HCM-6, and HCM-14) harboring each a missense variant classified as a VUS and a second PTV or splice-site variant reported in ClinVar database and classified as P according to ACMG. Six patients with variants in the *MYBPC3* carrying one or more additional variants in minor HCM genes (*ACTN2* and *MYH6*) and in recent HCM-associated genes (*FHOD3*, *TRIM63*, and *SVIL*) (Table 1; see Figure 3).

**Figure 4:**
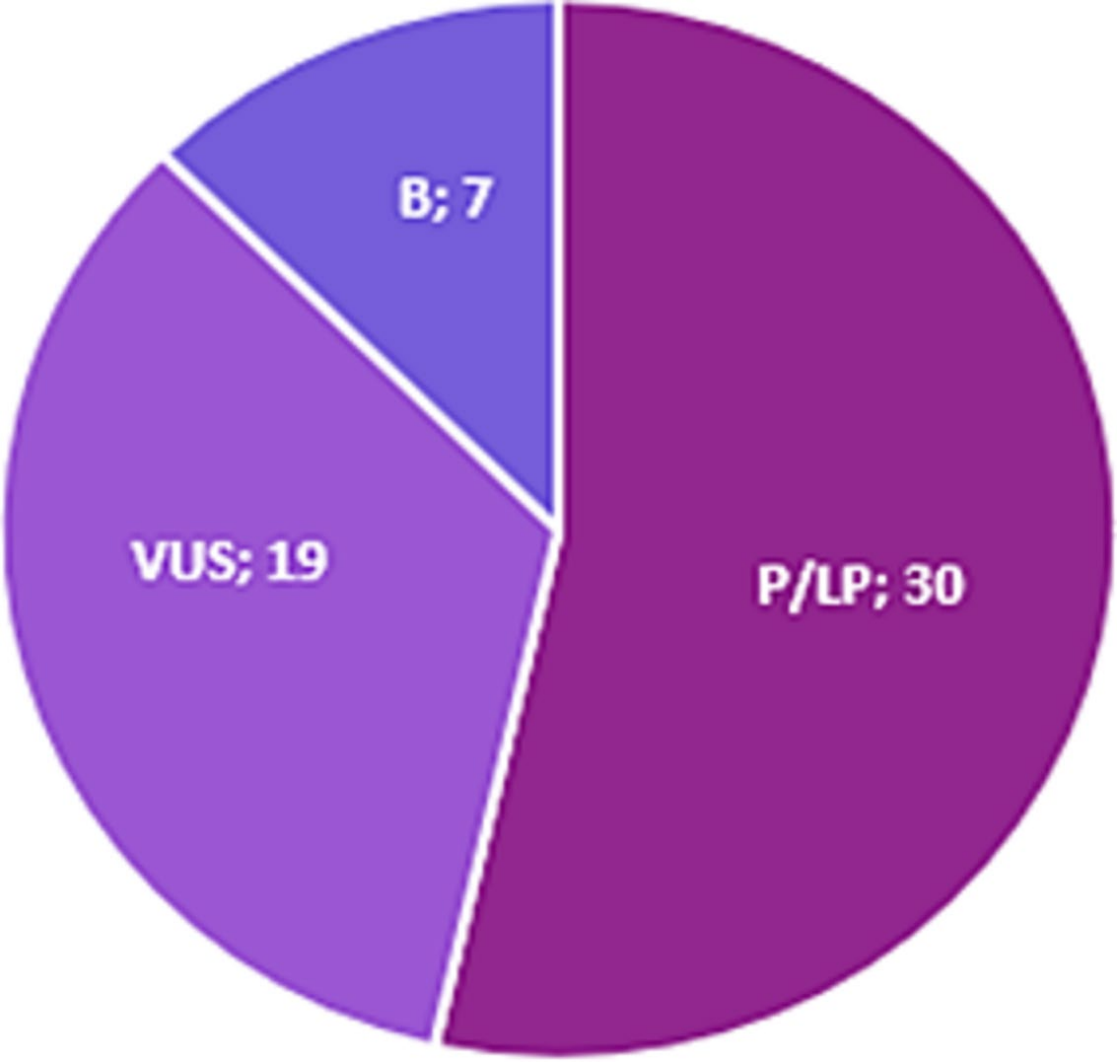
Proportion of *MYBPC3* and *MYH7* variants with P/LP ACMG classification. B, Benign; P, Pathogenic; LP, Likely Pathogenic; VUS, Variant of Uncertain Significance.

We identified 16 distinct variants in the *MYH7* gene, suggesting that these variants are more likely private. Importantly, in our case cohort, no PTV *MYH7* variant has been identified, and 8 out of the 16 missense variants are classified P and reported P or LP according to ACMG rules and ClinVar Database, respectively (Table 2). It should be noted that *MYBPC3* and *MYH7* are not only the most prevalent genes but also the most genes with P/LP classified variants following ACMG/AMP criteria (Figure 4). The remaining variants in this study are predicted to be deleterious through several tools. Each variant’s detailed *in silico* prediction is available in Supplementary File 2.

#### Troponin T2 and MLC genes: the minor sarcomeric genes

We identified a small proportion of patients carrying variants in *TNNT2*, *MYL2*, and *MYL3* genes (*n* = 6; 3%) (Table 3). Two patients carried missense variants in *TNNT2* (p.Val95Met and p.Lys283Glu), reported in ClinVar as LP and P, respectively. Furthermore, we identified four variants in *MYL2* (*n* = 1) and *MYL3* (*n* = 3) genes, one of which is at a homozygous state (*MYL3* p.Ala57Asp) with the conflicting interpretation of pathogenicity in ClinVar (Table 4).

All the variants in troponin T2 and MLC genes are unique.

### Variant identification among minor HCM genes

Several additional genes are consistently reported as HCM-causing genes encoding sarcomeric and non-sarcomeric proteins and contributing to a small proportion of HCM genetic etiology (13). In our reanalysis, we identified variants in 12 HCM minor genes. Rare and deleterious variants in *FLNC*, *MYH6*, *MYPN*, and *ACTN2* genes accounted for the majority of the prioritized variants among these genes (Table 5).

**Table 5:** Variants identified in minor HCM genes.

Patient ID	Genotype	Variant type	AA change	rs ID	CADD	ACMG/ClinVar	GnomAD-AF	Additional variant
Variants identified in the *FLNC* gene (transcript NM_001458, MANE select)								
HCM-92	Het	Missense	Ex4:p.(Ala280Thr)	rs778751919	34	VUS/VUS	0.00002004	Unique variant
HCM-93	Het	Missense	Ex13:p.(Asp693Ala)	rs34972246	28.9	VUS/LB	0.003442	Unique variant
HCM-67	Het	Missense	Ex23:p.(Arg1341Gln)	rs149641783	27.5	VUS/Conflicting	0.0009425	*MYH6*: p.(Gln277His)
HCM-94	Het	Missense	Ex28:p.(Ala1588Gly)	rs148545460	27.5	VUS/Conflicting	0.0001603	Unique variant
HCM-95	Het	Missense	Ex28:p.(Ser1624Leu)	rs879255639	34	VUS/Conflicting	0.00003189	Unique variant
HCM-66	Het	Missense	Ex30:p.(Arg1719Cys)	rs773260834	34	VUS/VUS	0.000008022	*MYH7*: p.(Glu1829Lys)
HCM-96	Het	Missense	Ex34:p.(Arg1860Cys)	rs181067717	34	VUS/LB	0.005209	Unique variant
HCM-97	Het	Missense	Ex38:p.(Gly2106Val)	NA	28.6	NA	NA	Unique variant
HCM-98	Het	Missense	Ex38:p.(Tyr2108Asn)	NA	28.6	NA	NA	Unique variant
HCM-99	Het	Missense	Ex40:p.(Gly2199Arg)	rs368977589	23.3	VUS/Conflicting	0.00003239	Unique variant
HCM-100	Het	Missense	Ex42:p.(Arg2364His)	rs201672146	25.7	VUS/LB	0.001724	*MYPN*: p.(Pro1112Leu)
HCM-101	Het	Missense	Ex42:p.(Arg2364His)	rs201672146	25.7	VUS/LB	0.001724	Unique variant
HCM-102	Het	Missense	Ex47:p.(Lys2637Gln)	rs767794768	20.9	VUS/VUS	0.00006479	Unique variant
Variants identified in the *MYH6* gene (Transcript NM_002471, MANE select)								
HCM-67	Het	Missense	Ex10:p.(Gln277His)	rs140660481	21.9	VUS/Conflicting	0.0003677	*FLNC*: p.(Arg1341Gln)
HCM-68	Het	Missense	Ex21:p.(Arg860His)	rs115845031	24.9	B /Conflicting	0.001778	Unique variant
HCM-69	Het	Missense	Ex22:p.(Glu932Lys)	NA	34	VUS/NA	NA	Unique variant
HCM-70	Het	Missense	Ex23:p.(Ala1004Ser)	rs143978652	22.9	LP/Conflicting	0.0009651	Unique variant
HCM-71	Het	Missense	Ex28:p.(Glu1295Gln)	rs34935550	29.1	LB/B	0.003167	Unique variant
HCM-72	Het	Missense	Ex28:p.(Glu1295Gln)	rs34935550	29.1	LB/B	0.003167	Unique variant
HCM-40	Het	Missense	Ex32:p.(Arg1532Leu)	rs34330111	34	VUS/Conflicting	0.0002794	*MYBPC3*: p.(Ala833Val) *SVIL*: p.(Thr1630Ser)
HCM-52	Het	Missense	Ex32:p.(His1526Arg)	NA	25.5	NA	NA	*MYH7*: p.(Asn232Ser) *CAV3*: p.(Thr78Met)
Variants identified in the *MYPN* gene (transcript NM_032578, MANE select)								
HCM-124	Het	Missense	Ex2:p.(Gln193Arg)	rs573684358	23.0	LB/VUS	0.0002188	Unique variant
HCM-100	Het	Missense	Ex17:p.(Pro1112Leu)	rs71534278	27.3	VUS/VUS	0.003060	*FLNC*: p.(Arg2364His)
HCM-125	Het	Missense	Ex17:p.(Pro1112Leu)	rs71534278	27.3	VUS/VUS	0.003060	Unique variant
HCM-126	Het	Missense	Ex17:p.(Pro1112Leu)	rs71534278	27.3	VUS/VUS	0.003060	Unique variant
HCM-127	Het	Missense	Ex17:p.(Ala1141Thr)	rs150404143	27.7	B/B	0.001085	*JPH2*: p.(Phe221Leu)
HCM-61	Het	Missense	Ex18:p.(Asp1208Gly)	NA	29.7	NA	NA	*MYH7*: p.(Arg787Cys) *VCL*: p.(His636Arg)
Variants identified in the *ACTN2* gene (transcript NM_001103, MANE select)								
HCM-9	Het	Missense	Ex10:p.(Thr347Met)	rs727504590	33	B/Conflicting	0.0001343	*MYBPC3*: p.(Arg238Cys)
HCM-121	Het	Missense	Ex12:p.(Arg438Trp)	rs563171274	34	VUS/Conflicting	0.00001194	Unique variant
HCM-116	Het	Missense	Ex12:p.(Val458Met)	rs895000018	33	LB/LB	0.000003985	*TRIM63:* p.(Gln247*)
HCM-122	Het	Missense	Ex17:p.(Lys694Asn)	rs748034053	24.7	LB/Conflicting	0.00003182	Unique variant
HCM-123	Het	Missense	Ex21:p.(Arg851His)	rs201335965	34	B/Conflicting	0.00004601	Unique variant
Variants identified in the *ALPK3* gene (transcript NM_020778, MANE select)								
HCM-86	Het	Missense	Ex5:p.(Tyr497Cys)	rs116077141	25.6	B/B	0.001299	Unique variant
HCM-87	Het	Missense	Ex7:p.(Arg1483Trp)	rs370816513	34	VUS/VUS	0.00002784	Unique variant
HCM-88	Het	Missense	Ex7:p.(Gly1522Asp)	NA	24.4	NA	NA	Unique variant
HCM-76	Het	Missense	Ex14:p.(Arg1907Gln)	rs116585466	32	VUS/VUS	0.0001469	*MYLK2*: p.(Gly89Asp)
Variants identified in the *CSRP3* gene (transcript NM_003476, MANE select)								
HCM-81	Het	Missense	Ex3:p.(Trp4Arg)	rs45550635	26.3	B/Conflicting	0.002285	Unique variant
HCM-82	Het	Missense	Ex3:p.(Trp4Arg)	rs45550635	26.3	B/Conflicting	0.002285	*SVIL*: p.(Thr1630Ser)
HCM-83	Het	Missense	Ex3:p.(Phe30Leu)	NA	32	VUS/VUS	NA	Unique variant
HCM-84	Het	Missense	Ex5:p.(Arg100His)	rs138218523	24.9	B/Conflicting	0.001323	Unique variant
Variants identified in the *MYLK2* gene (transcript NM_033118, MANE select)								
HCM-75	Het	Missense	Ex3:p.(Ala87Thr)	rs753089175	22.8	VUS/VUS	0.00003595	Unique variant
HCM-76	Het	Missense	Ex3:p.(Gly89Asp)	rs115398036	23	B/B	0.004242	*ALPK3*: p.(Arg1907Gln)
HCM-63	Het	Missense	Ex11:p.(Ser510Leu)	rs907738836	27.9	NA	NA	*MYH7*: p.(Arg869His) *TRIM63*: p.(Cys75Tyr)
Variants identified in the *NEXN* gene (transcript NM_144573, MANE select)								
HCM-89	Het	Missense	Ex9:p.(Glu332Ala)	rs201763096	23.2	B/B	0.004068	Unique variant
HCM-90	Het	Missense	Ex9:p.(Glu332Ala)	rs201763096	23.2	B/B	0.004068	Unique variant
Variants identified in the *CAV3* gene (transcript NM_033337, MANE select)								
HCM-52	Het	Missense	Ex2:p.(Thr78Met)	rs72546668	23.7	NA	0.002667	*MYH7*: p.(Asn232Ser) *MYH6*: p.(His1526Arg)
HCM-91	Het	Missense	Ex2:p.(Thr78Met)	rs72546668	23.7	NA	0.002667	*VCL:* p.(Arg285Cys)
Variants identified in the *VCL* gene (transcript NM_014000, MANE select)								
HCM-91	Het	Missense	Ex7:p.(Arg285Cys)	rs757517552	34	VUS/VUS	0.00001591	*CAV3:* p.(Thr78Met)
HCM-61	Het	Missense	Ex14:p.(His636Arg)	rs71579374	23.9	VUS/Conflicting	0.001485	*MYH7*: p.(Arg787Cys) *MYPN*: p.(Asp1208Gly)
Variants identified in the *TCAP* gene (transcript NM_003673, MANE select)								
HCM-85	Het	Missense	Ex2:p.(Met71Thr)	rs143465226	23	VUS/NA	0.00001669	Unique variant
Variants identified in the *JPH2* gene (transcript NM_020433, MANE select)								
HCM-127	Het	Missense	Ex2:p.(Phe221Leu)	rs558770240	23.2	B/Conflicting	0.001931	*MYPN*: p.(Ala1141Thr)

AA, amino acid; ACMG, American College of Medical Genetics and Genomics; Het, heterozygous; B, benign; P, pathogenic; LP, likely pathogenic; VUS, variant of uncertain significance; NA, no data available; Conflicting, conflicting interpretations of pathogenicity; CADD, combined annotation-dependent depletion scores according to CADD model v1.3; GnomAD-AF, allele frequency according to gnomAD V2.1.

Ten patients were found to carry unique variants in the *FLNC* gene, and three were digenic (HCM-67, HCM-66, and HCM-100) harboring an additional variant in the *MYH6*, *MYH7*, and *MYPN* genes, respectively. Unique variants in the *MYH6* gene were identified in five patients. Three out of six patients with *MYPN* variants carried the same p.Pro1112Leu variant.

Less unique variants were detected in the remaining minor genes *ALPK3*, *CSRP3*, *MYLK2*, *CAV3*, *VCL*, and *JPH2* genes. Of note, variants in *NEXN* (p.Glu332Ala) and *TCAP* (p.Met71Thr) are unique.

#### Emerging HCM genes: *TRIM63*, *FHOD3*, and *SVIL*

The main goal of this reanalysis was to identify variants within the recently associated genes not included in the initial HYPERGEN analysis. Three genes were identified toward this goal*TRIM63*, *FHOD3*, and *SVIL*.

##### *TRIM63* gene

**Figure 5:**
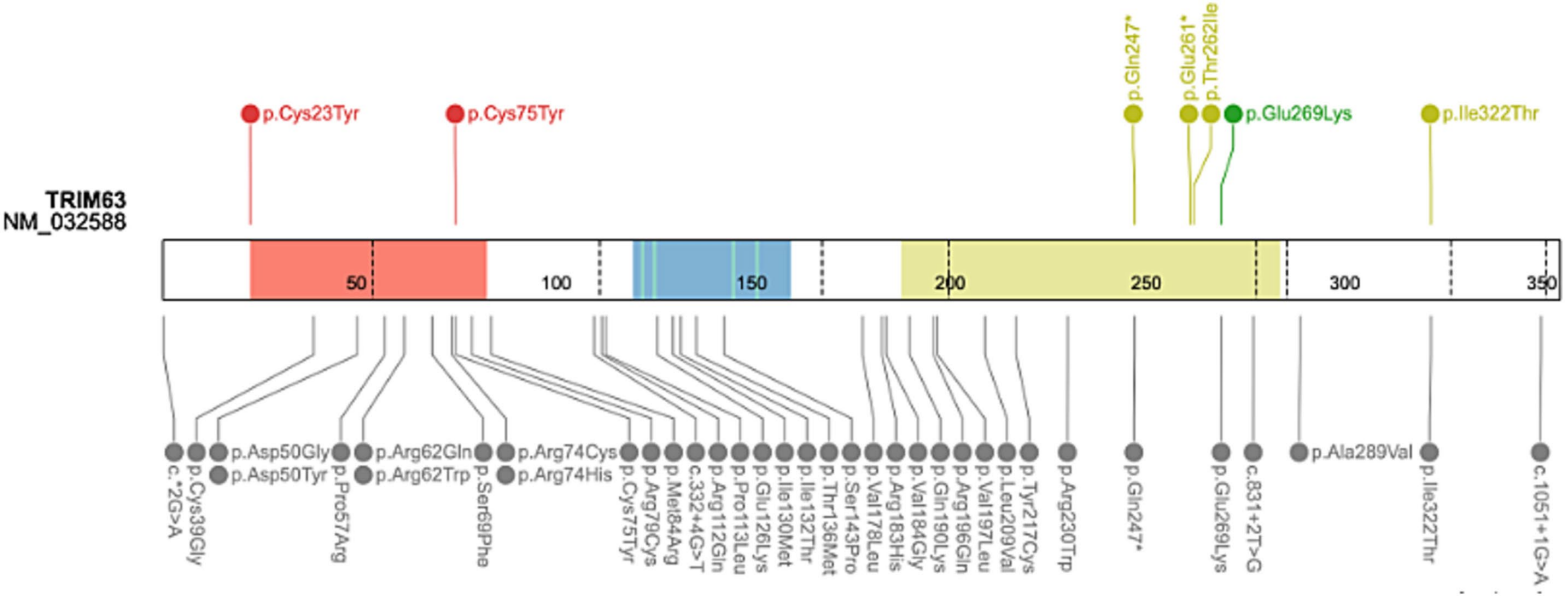
Schematic representation of the *TRIM63* gene. Upside: Rare variants identified in HYPERGEN cohort: Green: B/LB; Yellow: VUS; Red: LP/P; Downside: Variants reported in Clinvar database (clinical significance at last reviewed): Grey: VUS and conflicting interpretations of pathogenicity; Black: LP/P; Protein domains: Red = RING-finger domain (Zn-finger of 40 to 60 residue); Blue = B-Box-type zinc finger; Green = Zn2+ binding site; Yellow = Inter-Src homology 2 (iSH2) helical domain of Class IA Phosphoinositide 3-kinase Regulatory subunits. Figure created with ProteinPaint (https://proteinpaint.stjude.org/).

The *TRIM63* gene was associated with an autosomal recessive form of HCM (40, 41). In this study, we identified variants within the *TRIM63* gene in 12 patients. Three patients presented homozygous variants for *TRIM63* gene, and 6 variants were unique (Table 6). Indeed, the missense variants (p.Cys23Tyr and p.Cys75Tyr) were identified in patients at the heterozygous and homozygous states. Similarly, the stop variant (p.Gln247*) was found at the homozygous state in one patient and heterozygous for two other patients. According to ACMG/AMP classification, the missense variants Cys23Tyr and Cys75Tyr are LP. The *TRIM63* p.Gln247* stop variant is reported in ClinVar with conflicting interpretations of pathogenicity. A second stopgain variant (p.Glu261*) was found in a single patient (Figure 5). Of note, PTVs were identified only in *MYBPC3* and *TRIM63* genes.

**Table 6:** Variants identified in the *TRIM63* gene (transcript NM_032588, MANE select).

Patient ID	Genotype	Variant type	AA change	rs ID	CADD	ACMG/ClinVar	GnomAD-AF	Additional variant
HCM-44	Het	Missense	Ex1:p.(Cys23Tyr)	rs754874432	31	LP/NA	0.00003182	*MYBPC3*:p.(Arg1022Pro) *MYH7*: p.(His1185Gln)
HCM-111	Hom	Missense	Ex1:p.(Cys23Tyr)	rs754874432	31	LP/NA	0.00003182	*SVIL*: p.(Ser1414Thr)
HCM-112	Hom	Missense	Ex2:p.(Cys75Tyr)	rs200811483	31	LP/Conflicting	0.00009952	Unique variant
HCM-113	Het	Missense	Ex2:p.(Cys75Tyr)	rs200811483	31	LP/Conflicting	0.00009952	Unique variant
HCM-63	Het	Missense	Ex2:p.(Cys75Tyr)	rs200811483	31	LP/Conflicting	0.00009952	*MYH7*: p.(Arg869His) *MYLK2*: p.(Ser510Leu)
HCM-114	Hom	Stopgain	Ex5:p.(Gln247*)	rs148395034	38	VUS/Conflicting	0.0006787	Unique variant
HCM-115	Het	Stopgain	Ex5:p.(Gln247*)	rs148395034	38	VUS/Conflicting	0.0006787	Unique variant
HCM-116	Het	Stopgain	Ex5:p.(Gln247*)	rs148395034	38	VUS/Conflicting	0.0006787	*ACTN2*: p.(Val458Met)
HCM-117	Het	Stopgain	Ex5:p.(Glu261*)	rs149312738	40	VUS/NA	0.00001416	Unique variant
HCM-118	Het	Missense	Ex5:p.(Glu269Lys)	rs61749355	26.4	LB/NA	0.003159	Unique variant
HCM-119	Het	Missense	Ex5:p.(Thr262Ile)	rs889710255	25.6	VUS/NA	0.000003982	*SVIL*: p.(Glu1286Lys)
HCM-120	Het	Missense	Ex7:p.(Ile322Thr)	rs368532655	23.6	VUS/VUS	0.00001989	*SVIL*: p.(Val1490Ala)

AA, amino acid; ACMG, American College of Medical Genetics and Genomics; Het, heterozygous; Hom, homozygous; LB, likely benign; LP, likely pathogenic; VUS, variant of uncertain significance; NA, no data available; Conflicting, conflicting interpretations of pathogenicity; CADD, combined annotation-dependent depletion scores according to CADD model v1.3; GnomAD-AF, allele frequency according to gnomAD V2.1.

Clinical data were gathered for some patients with *TRIM63* variants. Two patients have septal hypertrophy and normal left ventricular ejection fraction (LVEF) (HCM-44 and HCM-113). Two patients with homozygous *TRIM63* variants (HCM-112 and HCM-114) have apical hypertrophy. The patient (HCM-117) with the stop variant p.Glu261* has a severe biventricular HCM with LVEF = 49% and mild aortic regurgitation.

##### *FHOD3* gene

**Figure 6:**
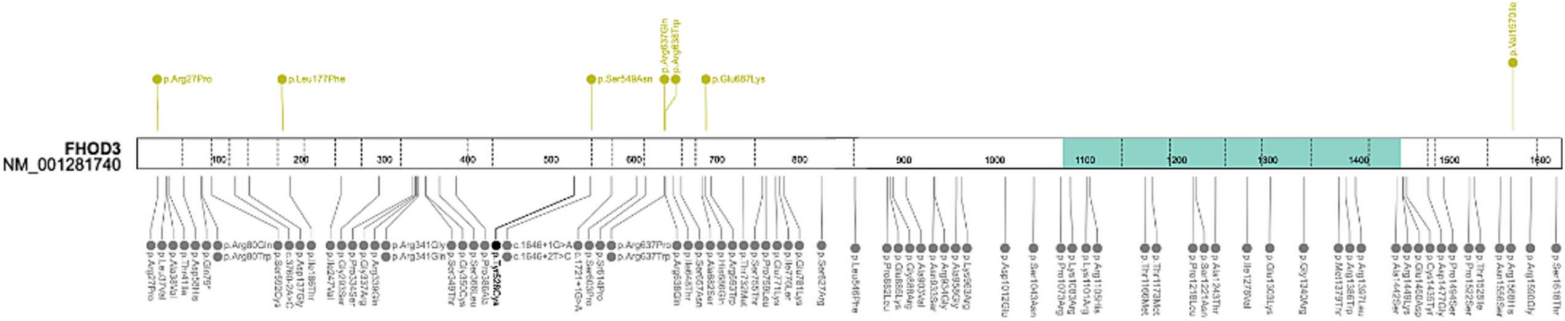
Schematic representation of the *FHOD3* gene. Upside: Rare variants identified in HYPERGEN cohort: Green = B/LB; Yellow = VUS; Red = LP/P; Downside: Variants reported in ClinVar database (clinical significance at last reviewed): Gray = VUS and conflicting interpretations of pathogenicity; Black = LP/P; protein domain: Green = Formin Homology 2 Domain. Figure was created with ProteinPaint (https://proteinpaint.stjude.org/).

The *FHOD3* locus is one of the most vital signals for HCM in genome-wide association study (GWAS) studies (42). Pathogenic variants are mainly located in two regions in the FHOD3 diaphanous inhibitory domain (exon 12) and the coiled–coil domain (exons 15 and 16). Moreover, it has been demonstrated that exons 11 and 12 are crucial for MybpC-mediated localization of the FHOD3 protein to the sarcomeric C-zone (12, 43–45). Our reanalysis further strengthens these associations by the identification of 3 missense variants in exon 12 and 15 in 6 patients of the HYPERGEN cohort. In total, 7 rare variants were prioritized in the *FHOD3* gene in 10 patients (Figure 6). The majority of variants are unique except for two patients (HCM-48 and HCM-27) with the recurrent *FHOD3* p.Arg637Gln variant. Both patients carried *MYBPC3* variants, p.(Cys1202Leufs*35) and p.(Arg597Gln), respectively (Table 7).

**Table 7:** Variants identified in the *FHOD3* gene (transcript NM_001281740, MANE select).

Patient ID	Genotype	Variant type	AA change	rs ID	CADD	ACMG/ClinVar	GnomAD-AF	Additional variant
HCM-103	Het	Missense	Ex1:p.(Arg27Pro)	rs755501978	33	VUS/VUS	0.0001619	Unique variant
HCM-104	Het	Missense	Ex6:p.(Leu177Phe)	rs781645381	26.8	VUS/NA	NA	Unique variant
HCM-105	Het	Missense	Ex12:p.(Ser549Asn)	NA	21.1	NA	NA	Unique variant
HCM-106	Het	Missense	Ex15:p.(Arg637Gln)	rs151313792	31	VUS/LB	0.001728	Unique variant
HCM-48	Het	Missense	Ex15:p.(Arg637Gln)	rs151313792	31	VUS/LB	0.001728	*MYBPC3:* p.(Cys1202Leufs*35)
HCM-27	Het	Missense	Ex15:p.(Arg637Gln)	rs151313792	31	VUS/LB	0.001728	*MYBPC3*: p.(Arg597Gln)
HCM-107	Het	Missense	Ex15:p.(Arg637Gln)	rs151313792	31	VUS/LB	0.001728	Unique variant
HCM-108	Het	Missense	Ex15:p.(Arg638Trp)	rs141148037	35	VUS/NA	0.0004252	*SVIL*: p.(Glu291Lys)
HCM-109	Het	Missense	Ex17:p.(Glu687Lys)	rs778872098	24.8	VUS/NA	0.000007986	Unique variant
HCM-110	Het	Missense	Ex28:p.(Val1570Ile)	rs201824593	33	VUS/NA	0.0007425	Unique variant

AA, amino acid; ACMG, American College of Medical Genetics and Genomics; Het, heterozygous; LB, likely benign; VUS, variant of uncertain significance; NA, no data available; CADD, combined annotation-dependent depletion scores according to CADD model v1.3; GnomAD-AF, allele frequency according to gnomAD V2.1.

We had access to the clinical description of one patient (HCM-104) with *FHOD3* variant (p.Leu177Phe). A definite HCM diagnosis was made at the age of 13 years. The patient had a concentric HCM with biventricular dilation. At the age of 31 years, his LVEF = 71% and RVEF = 53%.

##### *SVIL* gene

**Figure 7:**
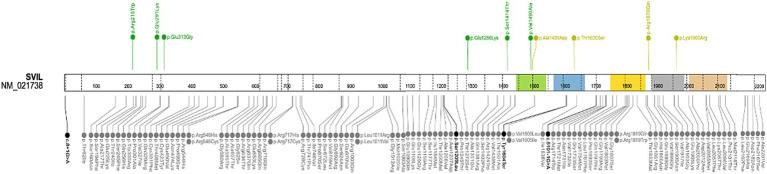
Schematic representation of the *SVIL* gene. Upside: Rare variants identified in HYPERGEN cohort: Green = B/LB; Yellow = VUS; Downside: Variants reported in ClinVar database (clinical significance at last reviewed): Gray = VUS and conflicting interpretations of pathogenicity; Black = LP/P; Protein domains: Green, blue, yellow, gray, and brown = gelsolin-like domains. Figure was created with ProteinPaint (https://proteinpaint.stjude.org/).

Recently, the *SVIL* gene was associated with HCM (46, 47). Thus, we performed a gene-targeted analysis for the *SVIL* gene. No PTV or homozygous variants were found in patients of the HYPERGEN cohort. Nevertheless, we identified 10 missense *SVIL* variants in 13 patients (Table 8; Figure 7). The prioritized variants are absent in 1920 control alleles. Five variants are unique. To better characterize the clinical presentation of *SVIL* variant carriers, cardiac and extracardiac features were gathered for 7 patients, two women and 5 men (Table 9). The age at onset of women patients was 29 and 27 years, one of them in the postpartum period. Moreover, the patient with *SVIL*: p.(Arg215Trp) had severe scoliosis with permanent bracing and muscle fasciculations. Three patients had aortopathies including a bicuspid aortic valve with severe regurgitation, isolated ectasia of the Valsalva sinus, and degenerative aortic insufficiency. A consistent pattern of fibrosis localization was noted in these patients in the septum and LV apex. Magnetic resonance imaging (MRI) findings showed significant myocardial fibrosis for the majority of patients with intramyocardial delayed contrast in the inferior and lateral walls (Table 9).

**Table 8:** Variants identified in the *SVIL* gene (transcript NM_021738, MANE select).

Patient ID	Genotype	Variant type	AA change	rs ID	CADD	ACMG/ClinVar	GnomAD-AF	Additional variant
HCM-129	Het	Missense	Ex6:p.(Arg215Trp)	rs74132329	26.2	B/B	0.003420	Unique variant
HCM-108	Het	Missense	Ex7:p.(Glu291Lys)	rs372012664	30	B/NA	0.00004242	*FHOD3*: p.(Arg638Trp)
HCM-130	Het	Missense	Ex8:p.(Glu313Gly)	rs138539716	25.7	B/NA	0.004283	Unique variant
HCM-119	Het	Missense	Ex21:p.(Glu1286Lys)	rs145704372	27.6	LB/NA	0.0003	*TRIM63*: p.(Thr262Ile)
HCM-111	Het	Missense	Ex23:p.(Ser1414Thr)	rs144661370	29.6	B/LB	0.001681	*TRIM63*: p.(Cys23Tyr)
HCM-134	Het	Missense	Ex23:p.(Ser1414Thr)	rs144661370	29.6	B/LB	0.001681	Unique variant
HCM-62	Het	Missense	Ex23:p.(Ser1414Thr)	rs144661370	29.6	B/LB	0.001681	*MYH7:* p.(Gly768Arg)
HCM-120	Het	Missense	Ex24:p.(Val1490Ala)	rs899379947	29.2	B/NA	0.00002477	*TRIM63*: p.(Ile322Thr)
HCM-39	Het	Missense	Ex25:p.(Ala1496Asp)		32			*MYBPC3*: p.(Trp792ValfsTer41)
HCM-40	Het	Missense	Ex27:p.(Thr1630Ser)	rs28451028	22.7	VUS/NA	0.004201	*MYBPC3*: p.(Ala833Val) *MYH6*: p.(Arg1532Leu)
HCM-82	Het	Missense	Ex27:p.(Thr1630Ser)	rs28451028	22.7	VUS/NA	0.004201	*CSRP3:* p.(Trp4Arg)
HCM-138	Het	Missense	Ex31:p.(Arg1870Gln)	rs762138005	35	VUS/NA	0.00001065	Unique variant
HCM-139	Het	Missense	Ex33:p.(Lys1960Arg)		19		0.000003976	Unique variant

AA, amino acid; ACMG, American College of Medical Genetics and Genomics; Het, heterozygous; B, benign; VUS, variant of uncertain significance; NA, no data available; CADD, combined annotation-dependent depletion scores according to CADD model v1.3; GnomAD-AF, allele frequency according to gnomAD V2.1.1.

**Table 9:** Clinical findings of *SVIL* variant carriers.

Patient ID	HCM-129	HCM-130	HCM-108	HCM-134	HCM-119	HCM-138	HCM-139
Gender	Female	Female	Male	Male	Male	Male	Male
Age at onset	29 years (postpartum period)	27 years	40 years	29 years	48 years	24 years	60 years
Initial symptoms	NA	Asthenia and dyspnea since childhood	SCV (VF)	Asthenia	A symptomatic	Asymptomatic	Syncopes during effort
Muscular abnormalities/deformities	Severe scoliosis with permanent bracing, muscle fasciculations	Isolated back pain	Not checked	None	Not reported by the patient/not checked	Not reported by the patient/not checked	Not reported by the patient/not checked
Arrhythmias	AF	NSVT	AF and VT/VF	AF	None	None	None
LV thickness TTE/MRI	26/19 mm	32/22 mm	31/30 mm	23/25 mm	19/19 mm	22/22 mm	16/15 mm
Fibrosis localization on MRI	LGE intramyocardial anteroseptal and apical	Severe intramyocardial LGE Apical and on the RV (rare)	Severe intramyocardial LGE inferoseptal and lateral	Severe intramyocardial LGE Septal	LGE intramyocardial Septal and apical	LGE Not significant	LGE intramyocardial anteroseptal
LVEF (%) TTE/MRI	60/30	50/64	50/63	70/67	65/70	75/79	70/74
Aortic phenotype	Normal	Normal	Normal	Bicuspid aortic valve associated with dilation of Valsalva’s sinus (41 mm) and severe aortic regurgitation Aortic valve replacement	Isolated ectasia of Valsalva’s sinus (39 mm)	Normal	Degenerative Aortic insufficiency (mild) without aortic dilatation
CK (0–170)	150–200	100–200	315	150–900	95	CPK (39–308): 79	CPK (39–308): 161
Tropo T (<14)	29–34	15–21	32–35	Tropo Ic US (0,000 – 0,050): 0,197	13	Tropo Ic US (0,000 – 0,050): 0,023	Tropo Ic US (0,000 – 0,050): <0,006
NT-proBNP	1,100–3,000	2,350	300–900	BNP (0–100): 713	379	BNP (0–100): 70	BNP (0–100): 66
*SVIL* variant	*SVIL*: p.(Arg215Trp)	*SVIL*: p.(Glu313Gly)	*SVIL*: p.(Glu291Lys)	*SVIL*: p.(Ser1414Thr)	*SVIL*: p.(Glu1286Lys)	*SVIL:* p.(Arg1870Gln)	*SVIL*: p.(Lys1960Arg)
Additional variant	None	None	*FHOD3*: p.(Arg638Trp)	None	*TRIM63*: p.(Thr262Ile)	None	None
Extracardiac features	Sickle cell disease; Bilateral hips prothesis; Appendicitis Osteoporosis Type-2 diabetes Hyperuricemia Arterial hypertension Tuberculosis (15 years) Non-secreting adrenal benign tumor Glaucoma	Sleep apnea syndrome	NA	NA	NA	NA	NA
Neurological exam/findings	Muscular fasciculations	Mild paresthesia (nocturnal)	Normal	Not performed	Not performed	Not performed	Not performed

AF, atrial fibrillation; CK, creatine kinase; HCM, hypertrophic cardiomyopathy; LGE, late gadolinium enhancement; LVEF, left ventricular ejection fraction; RV, right ventricular; VF, Ventricular fibrillation; VT, ventricular tachycardia; MRI, magnetic resonance imaging; NSVT, non-sustained ventricular tachycardia; NA, not available; SCD, sudden cardiac death; TTE, transthoracic echocardiography.

Only seven variants of the HYPERGEN cohort were present in the control cohort with low allele frequencies (Table 10). Moreover, four out of these seven variants were found to have the highest allele frequencies in the European non-Finnish population in gnomAD.

**Table 10:** HYPERGEN variants found in the control cohort.

Variant	Allele count	Number of homozygotes	Allele number	AF in a French control cohort	MAF Highest population in gnomAD
*TCAP*: p.Met71Thr	2	0	1920	0.001	African/African American: 6.582e-5
*ALPK3*: p.Arg1483Trp	1	0	1920	5.2083e-4	European non-Finnish: 6.155e-5
*VCL*: p.His636Arg	1	0	1920	5.2083e-4	European Finnish: 0.0063
*FLNC*: p.Arg1860Cys	28	2	1920	0.0145	European non-Finnish: 0.0082
*TRIM63*: p.Glu269Lys	6	0	1920	0.0031	European non-Finnish: 0.0058
*TRIM63*: p.Ile322Thr	6	0	1920	0.0031	European non-Finnish: 4.397e-5
*MYPN*: p.Pro1112Leu	3	0	1920	0.0015	Ashkenazi Jewish: 0.0097

AF, allele frequency; MAF, minor allele frequency.

#### The genetic landscape of young HYPERGEN patients

**Figure 8:**
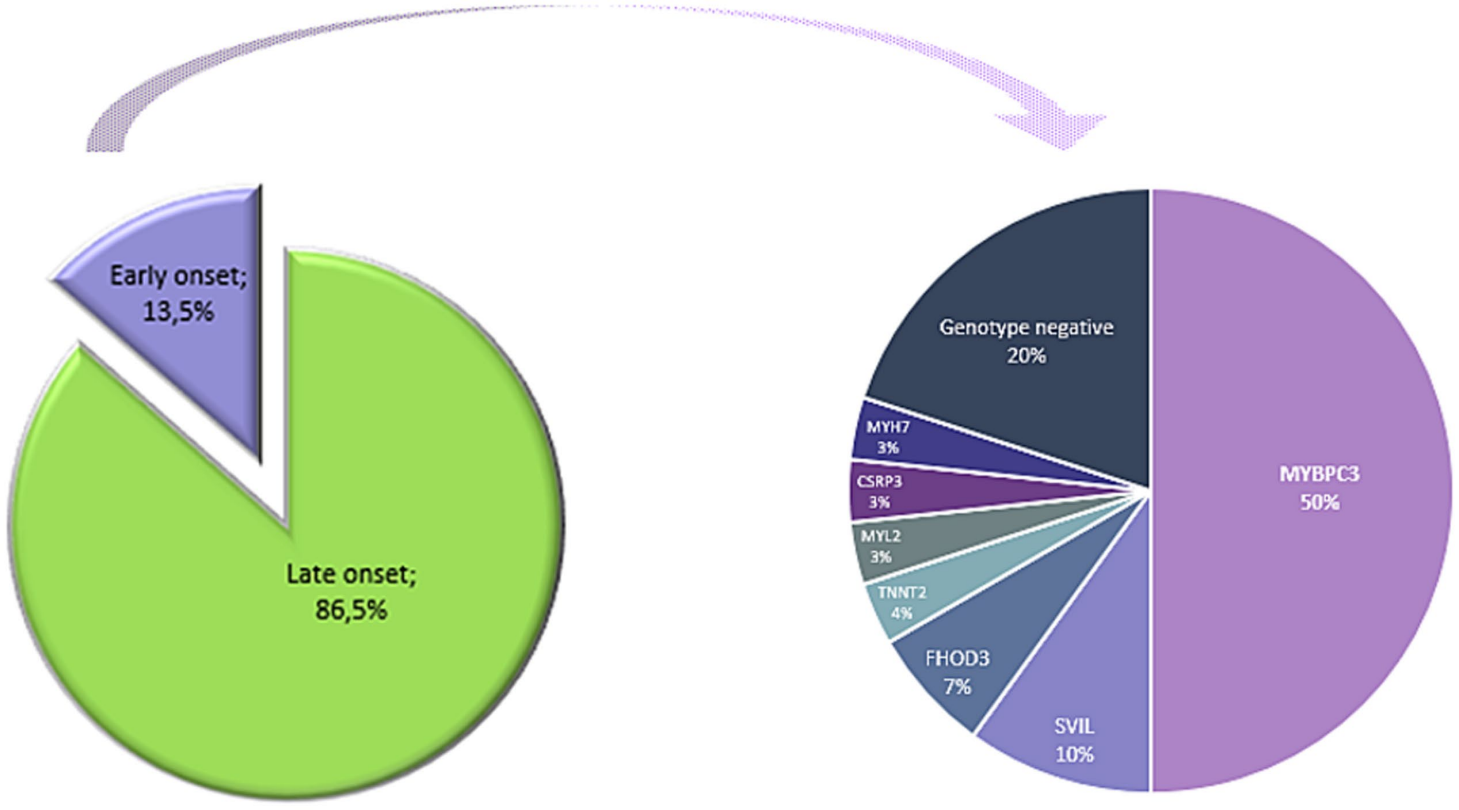
Percentages of gene variants distribution among young patients.

The HYPERGEN cohort included 27 patients with HCM occurring at a very young age or in early adulthood (13.5%). The HCM clinical diagnosis of the patients was definite before the age of 40 years. We sought to determine the genetic architecture of this young proportion. Indeed, sarcomeric genes were the most involved genes, as 50% of the identified variants are within the *MYBPC3* gene. Interestingly, variants in *SVIL* and *FHOD3* genes accounted for 10 and 7% of young HYPERGEN patients, respectively (Figure 8).

**Figure 9:**
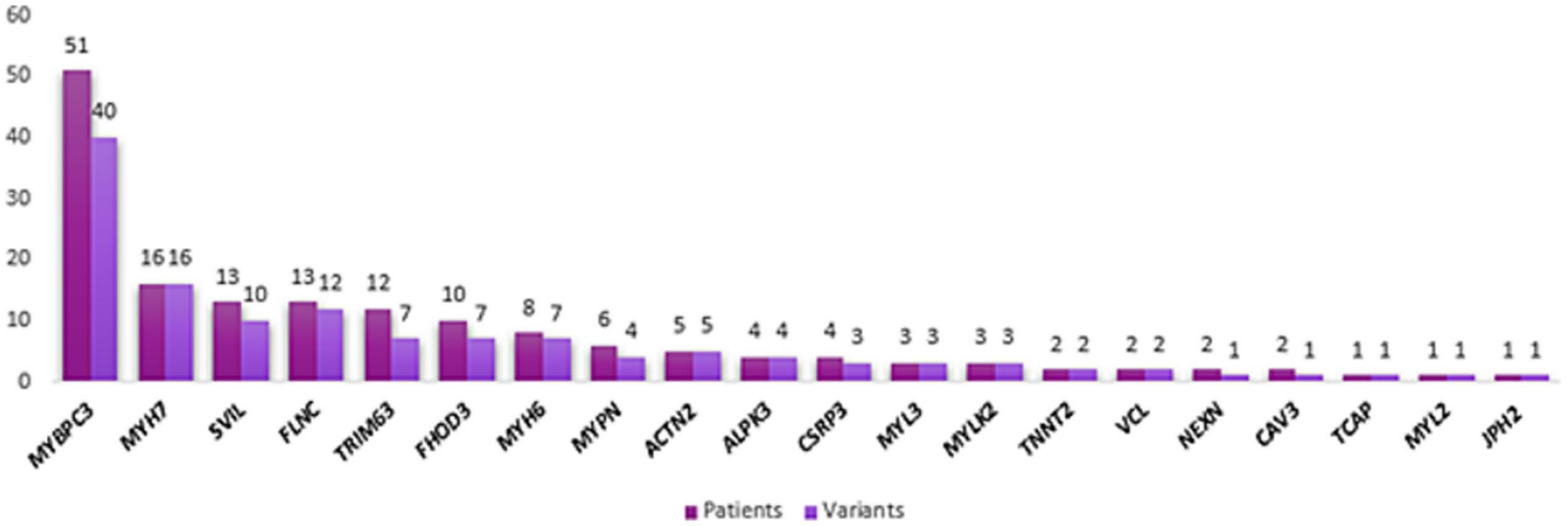
Genes implicated in the HYPERGEN cohort. The number of variants is indicated in dark purple, and the number of patients is indicated in light purple.

In summary, a total of 20 genes have been identified in the HYPERGEN cohort, with a significant implication of the *MYBPC3* gene, followed by *MYH7* and *SVIL* genes (Figure 9). According to ClinGen, 8 out of the 20 identified genes are classified with a robust association with HCM, 7 with disputed/limited evidence, and the *JPH2* gene with a moderate gene–disease validity. The *FLNC*, *CAV*, and *SVIL* are not curated for HCM, and the *FHOD3* gene is under curation (Table 11). However, those genes are reported in the literature and in the OMIM database in association with HCM.

**Table 11:** List of the identified genes in the HYPERGEN cohort and their matched ClinGen classification.

Gene symbol	Gene name	Gene-HCM validity classification	Inheritance	Gene–disease validity classification		Inheritance
*MYBPC3*	Myosin binding protein C3	Definitive (MONDO:0005045)	AD	Dilated cardiomyopathy (MONDO:0005021)	Limited	AD
				Arrhythmogenic right ventricular cardiomyopathy (MONDO:0016587)	Limited	
*MYH7*	Myosin heavy chain 7	Definitive (MONDO:0005045)	AD	Dilated cardiomyopathy (MONDO:0005021)	Definitive	AD
				MYH7-related skeletal myopathy (MONDO:0008050)	Definitive	
				Arrhythmogenic right ventricular cardiomyopathy (MONDO:0016587)	Limited	
				Congenital heart disease (MONDO:0005453)	Limited	
*MYL2*	MLC 2	Definitive (MONDO:0005045) Definitive (MONDO:0012112)	AD	Dilated cardiomyopathy (MONDO:0005021)	Limited	AD
				Arrhythmogenic right ventricular cardiomyopathy (MONDO:0016587)	No known disease relationship	
*MYL3*	MLC 3	Definitive (MONDO:0005045)	AD	Arrhythmogenic right ventricular cardiomyopathy (MONDO:0016587)	Limited	AD
				Dilated cardiomyopathy (MONDO:0005021)	Disputed	
*TNNT2*	Troponin T2, Cardiac Type	Definitive (MONDO:0005045)	AD	Dilated cardiomyopathy (MONDO:0005021)	Definitive	AD
				Arrhythmogenic right ventricular cardiomyopathy (MONDO:0016587)	No known disease relationship	
*MYLK2*	MLC Kinase 2	Disputed (MONDO:0005045)	AD	NA	NA	NA
*CSRP3*	Cysteine and glycine-rich protein 3	Definitive (MONDO:0005045)	SD	Dilated cardiomyopathy (MONDO:0005021)	Limited	AD
		Moderate (MONDO:0005045)	AD			
*MYH6*	Myosin heavy chain 6	Limited (MONDO:0005045)	AD	Dilated cardiomyopathy (MONDO:0005021)	Limited	AD
*ACTN2*	Actinin α2	Definitive (MONDO:0000591)	AD	NA	NA	NA
*ALPK3*	α-Kinase 3	Definitive (MONDO:0005045)	AR	NA	NA	NA
*VCL*	Vinculin	Disputed (MONDO:0005045)	AD	Dilated cardiomyopathy (MONDO:0005021)	Moderate	NA
*JPH2*	Junctophilin 2	Moderate (MONDO:0005045)	AD	Dilated cardiomyopathy (MONDO:0005021)	Moderate	SD
*NEXN*	Nexilin F-actin binding protein	Limited (MONDO:0005045)	AD	Dilated Ccrdiomyopathy (MONDO:0005021)	Moderate	AD
*MYPN*	Myopalladin	Disputed (MONDO:0005045)	AD	MYPN-related Myopathy (MONDO:0015023)	Definitive	AR
				Dilated cardiomyopathy (MONDO:0005021)	Limited	AD
*TCAP*	Titin-Cap	Disputed (MONDO:0005045)	AD	Limb–girdle muscular dystrophy (MONDO:0015152)	Definitive	AR
				Dilated cardiomyopathy (MONDO:0005021)	Limited	AD
*FLNC*	Filamin C	NA	NA	Dilated cardiomyopathy (MONDO:0005021)	Definitive	AD
				Myofibrillar myopathy 5 (MONDO:0012289)	Definitive	
*FHOD3*	Formin homology 2 domain containing 3	Under active curation		NA	NA	NA
*CAV3*	Caveolin 3	NA	NA	Caveolinopathy (MONDO:0016146)	Definitive	AD
				Long QT syndrome (MONDO:0002442)	Limited	
*TRIM63*	Tripartite motif containing 63	Moderate (MONDO:0005045)	AR	NA	NA	NA
		Disputed (MONDO:0005045)	AD			
*SVIL*	Supervillin	NA	NA	NA	NA	NA

AD: autosomal dominant; AR: autosomal recessive; HCM, hypertrophic cardiomyopathy; MLC, myosin light chain; NA, not available; SD: semidominant.

Our reanalysis yielded 16 novel variants, including four in the *MYBPC3* gene, three in *MYH7*, two variants were found in each of *MYH6* and *FLNC* genes, and one novel variant in *TNNT2*, *MYL2*, *MYPN*, *ALPK3*, and *CSRP3*, respectively.

Of note, considering only patients with unique variants in *TRIM63*, *FHOD3*, and *SVIL* genes, there was a 9% enhancement in variant identification following this reanalysis.

All the identified genes and their matched phenotypes in the OMIM database are summarized in Supplementary File 3.

## Discussion

Despite the advent of next-generation sequencing, approximately 50% of cases remain genotype-elusive. In this study, a reanalysis of exome data from 200 HCM patients was carried out 5 years after the initial analysis with the goal of refining the initial analysis, reporting all the relevant, prioritized variants, and particularly identifying rare variants within the novel HCM-associated genes.

In the present reanalysis, nearly 34% of patients were found to carry variants in *MYBPC3* and *MYH7* genes. This yield of sarc+ patients reached 37% when including patients with *TNNT2*, *MYL2*, and *MYL3* variants.

Haploinsufficiency is the primary mechanism driving HCM linked to *MYBPC3* (myosin binding protein C3) gene (38, 48). Thus, non-truncating variants (missense, in-frame indels, PTV predicted to escape non-sense mediated decay [NMD], and stop-loss) are prioritized whether they are predicted as pathogenic with high or low confidence. Our reanalysis yielded a total of 40 *MYBPC3* variants, including 24 missense, 4 splice-site, 6 stopgain, and 6 frameshift variants. The most recurrent and common HCM causal variant *MYBPC3*, p.Arg502Trp, was identified in only three patients (1.5%). This variant was found in 2.4% of the European-descent patients (15, 49–51). However, the pathogenic *MYBPC3*: c1928-2A > G variant was the most commonly identified variant in the HYPERGEN cohort (*n* = 7; 3.5%). Notably, these variants are unique except for one patient (HCM-5) harboring a second *MYBPC3* variant (p.Leu183Ile) (Table 1). Contrary to the *MYBPC3* gene, mostly known with haploinsufficiency and allelic imbalance as the underlying mechanism of the disease, the pathophysiology mechanism of *MYH7* (myosin heavy chain 7) missense variants is the dominant negative effect, which implies that the *MYH7* gene is tolerant to loss of function (LoF) variants (10, 38, 39, 52, 53). Thus, prioritizing the *MYH7* missense variant with a predicted deleterious impact according to different *in silico* algorithms may increase the identification rate of actionable variants. Moreover, gene regions in which rare and likely causal variants are significantly clustered; therefore, a new etiological fraction-based ACMG rule on rare missense *MYH7* variants was proposed to improve genetic testing yield in HCM (30). All the identified *MYH7* variants in this study are missense (*n* = 16) with 8 P/LP variants. Of note, variants in the *MYH7* gene were associated with significant LV hypertrophy, which is the hallmark of HCM and an unfavorable prognosis compared to patients carrying variants in the other HCM genes (54).

We identified a small proportion of patients (3%) carrying variants in *TNNT2*, *MYL2*, and *MYL3* genes. Only two variants in the *TNNT2* gene were identified. The *TNNT2* gene encodes the cardiac troponin T2 which is a regulatory protein of the thin filament troponin complex in the sarcomere playing a crucial role in contractility function (39, 55). More than 30 *TNNT2* P/LP variants have been linked to HCM, with individual variants being unique and private to distinct families (55). Moreover, phenotypic variability was reported among patients carrying the same *TNNT2* variant (55). The MLCs are composed of a regulatory light chain (*MYL2*) and an essential light chain (*MYL3*). These genes contribute to the stability of myosin head and the regulation of cardiac contraction by phosphorylation and Ca^2+^ binding (52). Despite these genes being considered well-established HCM genes, their contribution to HCM etiology is limited. A study by Borrelli et al. estimated that the contribution of genes encoding sarcomere thin filaments does not exceed 5% (11). In our cohort, no high or moderate confidence variants were found in *ACTC1*, *TPM1*, *TNNC1*, or *TNNI3* genes. A recent study by Allouba et al. (56) showed that homozygous variants are more prevalent within *MYL2* and *MYL3* genes than within major sarcomeric HCM genes. In the HYPERGEN cohort, only one homozygous variant was identified (*MYL3*: p.Ala57Asp) in a patient with a family history of HCM.

Although HCM has been recognized as a monogenic disease for a long time, the wide utilization of high-throughput sequencing has demonstrated that it may be caused by the occurrence of more than one variant, particularly for sarc−patients. In this reanalysis, considering sarc+patients, we identified three patients with two variants within the *MYBPC3* gene, seven cases carrying digenic variants, and five patients carrying more than two variants (Tables 1, 2). In some sarc−cases, unique variants were found in genes encoding proteins located in the Z-disc, namely, *FLNC*, *ACTN2*, *CSRP3*, *TRIM63*, and *SVIL* (Tables 5–8). Of note, variants within *CAV3*, *VCL*, and *JPH2* genes were found along with additional sarcomeric and non-sarcomeric variants.

We identified three *CSRP3* variants, including the known p.Trp4Arg variant. The *CSRP3* gene, encoding cysteine and glycine-rich protein 3, is one of the non-sarcomeric HCM-associated genes with strong evidence for a primary pathogenic role in HCM (13). It plays different roles in mechanosensory functions and actin cytoskeleton assembly (12, 57). Functional analysis of *Csrp3* knock-in animals (*Csrp3^Trp4Arg/+^*) and (*Csrp3^Trp4Arg/Trp4Arg^*) showed an age-and gene dosage-dependent HCM and heart failure, characterized by a nearly complete loss of contractile function under catecholamine-induced stress. Moreover, *Cspr3* mRNA and protein levels were significantly decreased in the hearts of heterozygous and homozygous *Cspr3^Trp4Arg^* knock-in animals (57).

We identified five variants in the *ACTN2* gene, three of which were unique, and two patients were digenic and were carrying additional variants in *MYBPC3* and *TRIM63* genes. The *ACTN2* gene (major Z-disc cross-linking protein) is of particular interest as variants within this gene have been linked to diverse cardiac phenotypes such as HCM, dilated cardiomyopathy (DCM), LV non-compaction (LVNC), and SCD (Supplementary File 3) (58–61). Patients with *ACTN2* pathogenic variants showed no specific hypertrophy pattern, as septal, apical, concentric, and biventricular hypertrophy were reported. More importantly, patients with mild hypertrophy had severe complications such as resuscitated cardiac arrest and heart failure (12, 61–64).

A small number of variants was identified within the *ALPK3* gene (*n* = 4), 3 out of the 4 variants were unique. Of note, the *ALPK3* gene (*α*-protein kinase 3) was initially linked to an autosomal recessive form of severe pediatric mixed cardiomyopathy (HCM/DCM phenotype) (65–67). In 2020, the *ALPK3* gene reached a definitive classification of strong evidence for an autosomal recessive form of HCM with an infant onset (46). However, cases harboring heterozygous LoF variants were reported with mild-to-moderate phenotypes, and an autosomal dominant pattern of inheritance was subsequently associated with an adult-onset HCM (12, 46, 68, 69). Recently, rare missense *ALPK3* variants have been identified in Asian HCM patients (46, 70).

Three genes were recently associated with HCM and identified in this reanalysis: *TRIM63*, *FHOD3*, and *SVIL*. *TRIM63* is one of the rare genes recently described as a cause of HCM with autosomal-recessive inheritance. *TRIM63* encodes muscle-specific RING-finger protein 1 (MuRF1), a member of the ubiquitin ligases subfamily, such as MuRF-2 and MuRF-3 (71, 72). It is an E3 ubiquitin−protein that regulates the degradation of sarcomeric proteins such as Mybpc3 and Myh6 through ubiquitylation (12, 72). Homozygous and compound heterozygous rare variants in the *TRIM63* gene were linked to HCM. *TRIM63* variant carriers showed concentric LV hypertrophy, significant cardiac fibrosis, LV systolic dysfunction, and arrhythmias (12, 40, 41). Furthermore, systolic dysfunction and late gadolinium enhancement have been reported as characteristic features of *TRIM63*-associated cardiomyopathies (41). Rare missense variants in *TRIM63* at the heterozygous state were reported as genetic modifiers of HCM. Although the *TRIM63* missense variants had limited evidence of disease causality, *TRIM63* knockouts are likely to be associated with HCM, given their enrichment in HCM patients and their absence in gnomAD (12, 41). In our reanalysis, the two LP missense variants (p.Cys23Tyr and p.Cys75Tyr) were identified in 5 patients of the HYPERGEN cohort. The *TRIM63*: (p.Gln247*) stop variant was identified in three patients, one at the homozygous state and two patients were heterozygous. The p.Gln247* variant is reported in the ClinVar database with a conflicting interpretation of pathogenicity. Nevertheless, *in vitro* and *in vivo* functional studies showed near complete loss of auto-ubiquitination in cells transduced with the TRIM63Q247* lentiviral construct (12, 41). Several other *TRIM63* variants impair the ubiquitination of Trim63 substrates in adult cardiomyocytes. These findings implicate the impaired protein degradation as a pathophysiology mechanism of HCM (40, 41).

Of note, in the HYPERGEN cohort, five patients (2.5%) carried homozygous variants in *MYBPC3* (*n* = 1), *MYL3* (*n* = 1), and *TRIM63* (*n* = 3) genes.

The second recent gene identified in this reanalysis is the *FHOD3* gene. *FHOD3* encodes the cardiac formin homology 2 domain containing three proteins that localize in the thin filament of the sarcomere and promote actin filament polymerization in cardiomyocytes (44).

Rare pathogenic *FHOD3* variants were linked to HCM etiology through association analysis of 3,189 HCM patients and familial segregation studies (43). *FHOD3* variants associated with HCM are mostly non-truncating and disturb the diaphanous inhibitory domain of the protein. Patients carrying likely causative *FHOD3* variants showed mild-to-moderate LV hypertrophy and a 1% annual incidence of cardiovascular mortality (43). Additionally, *FHOD3*-HCM patients were diagnosed in adulthood (mean age 46.1 years), and two-thirds (66%) were men. The majority of patients (82%) had asymmetric septal hypertrophy (mean 18.8 ± 5 mm). LV ejection fraction <50% was present in 14% of the cohort and hypertrabeculation in 16% (43). Moreover, the cardiomyopathic phenotype of *cMyBP-C* null mice was aggravated by Fhod3 overexpression with a sarcomere integrity disruption. This phenotype was partially improved by a reduction in the Fhod3 protein levels, suggesting that Fhod3 has a damaging impact on cardiac function under *cMyBP-C* null conditions where Fhod3 is mis-localized (44). These findings suggest the likely contribution of Fhod3 to the pathogenesis of cMyBP-C-related cardiomyopathy and that Fhod3 is implicated in cardiac cMyBP-C-mediated regulation via direct interaction (44). In this study, rare missense *FHOD3* variants were identified in the hotspot exons (12 and 15), and two patients with the recurrent *FHOD3* p.Arg637Gln variant were digenic, carrying additional variants in the *MYBPC3* gene (p.Cys1202Leufs*35 and p.Arg597Gln).

More recently, the *SVIL* gene was associated with HCM (46, 47). The *SVIL* (Supervillin) encodes a multidomain actin and myosin-binding protein in the Z-disc (73, 74). Biallelic LoF *SVIL* variants were identified in patients with skeletal myopathy, mild LV hypertrophy, and smaller descending aortic diameter (75). Indeed, a 10.5-fold excess burden of *SVIL* rare PTV variants in HCM cases has been demonstrated in a recent GWAS (47). Patients harboring rare truncating *SVIL* variants were found to have an increased LV contractility in both obstructive and non-obstructive forms of HCM, as demonstrated by Mendelian randomization analyses (47). In one family, the *SVIL*: p.(Gln255*) variant was found in two affected cousins, providing some evidence of familial segregation (47). In this reanalysis, clinical data were gathered for 7 out of the 13 patients with *SVIL* variants (Table 9). Three patients harboring the *SVIL*: p.(Ser1414Thr), p.(Glu1286Lys), and p.(Lys1960Arg) variants presented an aortic phenotype including bicuspid aortic valve disease, isolated ectasia of Valsalva sinus, and mild aortic insufficiency. Overall, the majority of *SVIL* patients showed severe intramyocardial late gadolinium enhancement. One patient (HCM-108) experienced SCD, and another patient (HCM-139) had a history of syncopes during physical effort. Of note, the patient (HCM-108) carried an additional variant in the *FHOD3* gene (p.Arg638Trp). A severe scoliosis was diagnosed in one patient (HCM-129).

Interestingly, variants in *SVIL* and *FHOD3* genes accounted for 10 and 7%, respectively, of patients with early HCM onset. Patients with sarcomere mutations have been reported to show earlier adverse complications and a worse prognosis (76). Several published HCM cohorts, studies, and data provided by the Sarcomeric Human Cardiomyopathy Registry (SHaRe) have shown that cases with sarcomeric variants had an early onset of the disease and a more severe phenotype with malignant complications. Thus, patients diagnosed in early adulthood (<40 years) had more severe outcomes with many adverse complications compared to patients with late-onset HCM. Moreover, HCM young patients (20–29 years) had a 4-fold increase in the risk of death compared to the general population (76–78).

All the identified variants in this reanalysis were searched in a French control cohort, gathering 960 healthy individuals. Only seven variants were present (Table 10). Of note, *TCAP*: p.(Met71Thr), *ALPK3*: p.(Arg1483Trp), *FLNC*: p.(Arg1860Cys) and *MYPN*: p.(Pro1112Leu) variants were unique in HYPERGEN carriers. The *VCL*: p.(His636Arg) and *MYPN*: p.P (ro1112Leu) variants were found in Finnish and Ashkenazi Jewish populations, respectively, suggesting that these variants could be more prevalent in these bottleneck populations.

Although the identification of variants in genes lacking strong evidence for disease causality and/or VUS in minor HCM genes does not increase the clinically actionable genes/variants discovery, it sheds light on the possibility of a combinatorial joint effect where VUS variants may act as a small risk increasing genetic factor.

Gathering strong proof of disease causality remains challenging for many reasons, such as the unavailability of family members to undergo co-segregation and funding constraints for functional studies. Indeed, genes such as *ALPK3* and *FHOD3* have been considered disease-causing by large gene-centric case–control studies. This strategy is an effective approach to reaching the needed statistical power supporting gene–disease association (46). Furthermore, the publication of cases with variants of low to moderate evidence was recommended, as the identical variants may be detected in extended families with similar phenotypes by researchers interested in functional validation (46).

Presently, we aimed to reanalyze a cohort of 200 HCM patients by focusing on genes strongly associated with HCM while also investigating newly linked genes with limited or insufficient evidence of causality to determine their potential involvement in our cohort. Overall, our results strengthen the implication of *FHOD3*, *TRIM63*, and *SVIL* genes in HCM as minor genes. Variants in the *SVIL* gene, recently linked to HCM, were found in 13 HYPERGEN patients. More importantly, patients harboring *SVIL* variants presented a similar hypertrophic pattern (significant myocardial fibrosis for six out of the seven patients/apical for three patients), and aortopathy was reported for three patients. The findings from our study extend the clinical and genetic spectrum of *SVIL* gene carriers. While Tadros et al. (47) identified *SVIL* variants in HCM patients, our study reveals additional cases, underscoring the need for expanded genetic screening. This broader understanding can facilitate future investigations into the pathophysiological mechanisms associated with *SVIL* variants, potentially leading to improved risk stratification and management strategies for patients.

The assessing and reporting of the identified variants—particularly within the novel associated genes—may provide additional strength to the previously reported variants and identify novel variants that help an accurate classification and interpretation. This study contributes to defining the genetic landscape of French HCM patients, which facilitates cascade genetic testing in familial cases and ultimately could guide personalized treatment.

The main goal of this study was to assess and report actionable and high-confidence variants in known and emerging HCM genes. However, there are several limitations in our study, making an accurate estimation of the increased diagnostic yield challenging. This is mainly due to the absence of detailed genetic results from the initial analysis. In addition, detailed patient phenotyping and clinical data are lacking. Thus, genotype–phenotype correlations and risk stratification were difficult to achieve.

## Data Availability

The data presented in the study are deposited in the ClinVar database under the following link: https://www.ncbi.nlm.nih.gov/clinvar/submitters/508840/.

## Footnotes

^1^ https://www.clinicalgenome.org/

^2^ https://github.com/lindenb/gazoduc-nf/tree/aab8c83f47267e64a24a37bed686ec4f041003e6/workflows/gatk/gatk4gvcfs

^3^ https://doi.org/10.6084/m9.figshare.1425030.v1
