# Corrigendum: Exome sequencing data reanalysis of 200 hypertrophic cardiomyopathy patients: the HYPERGEN French cohort 5 years after the initial analysis

**DOI:** 10.3389/fmed.2025.1595049

**Published:** 2025-05-06

**Authors:** Hager Jaouadi, Victor Morel, Helene Martel, Pierre Lindenbaum, Lorcan Lamy de la Chapelle, Marine Herbane, Claire Lucas, Frédérique Magdinier, Gilbert Habib, Jean-Jacques Schott, Stéphane Zaffran, Karine Nguyen

**Affiliations:** ^1^Aix Marseille Université, INSERM, Marseille Medical Genetics (MMG), U1251, Marseille, France; ^2^Department of Medical Genetics, La Timone Hospital, AP-HM, La Timone Children's Hospital, Marseille, France; ^3^Department of Cardiology, La Timone Hospital, AP-HM, Marseille, France; ^4^Nantes Université, CHU Nantes, CNRS, INSERM, l'institut du Thorax, Nantes, France

**Keywords:** HCM, exome reanalysis, sarcomeric genes, non-sarcomeric genes, novel-associated genes, FHOD3, TRIM63, SVIL

In the published article, an author name was incorrectly written as Habib Gilbert. The correct spelling is Gilbert Habib.

In the published article, there was an error in affiliation 1. Instead of "Marseille Medical Genetics (MMG) U1251, Aix Marseille Université, INSERM, Marseille, France” it should be “Aix Marseille Université, INSERM, Marseille Medical Genetics (MMG), U1251, Marseille, France”.

In the published article, an author name was incorrectly written in the citation as “de la Chapelle LL”. The correct citation is “Lamy de la Chapelle L”.

The authors apologize for this error and state that this does not change the scientific conclusions of the article in any way. The original article has been updated.

