# Supplementary material for: Exome sequencing data reanalysis of 200 hypertrophic cardiomyopathy patients: the HYPERGEN French cohort 5 years after the initial analysis
Source: Front Med (Lausanne). 2024 Oct 31;11:1480947. doi: 10.3389/fmed.2024.1480947 (PMC11565434; doi:10.3389/fmed.2024.1480947)
Supplement: Supplementary file 1 [file Table_1.DOCX]

Sanger confirmation of the identified variants in the HYPERGEN cohort.

***MYBPC3* variants**


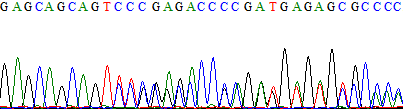


Patient ID: HCM-12

*MYBPC3*: c.913_914del:p.(Phe305ProfsTer27)


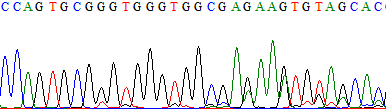


Patient ID: HCM-18

*MYBPC3*: c.1310delT:p.(Val437GlyfsTer13)


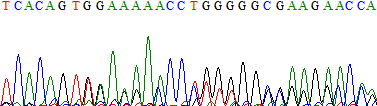


Patient ID: HCM-36

*MYBPC3*: c.2258dupT:p.(Lys754GlufsTer79)


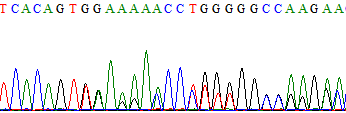


Patient ID: HCM-37

*MYBPC3*: c.2258dupT:p.(Lys754GlufsTer79)


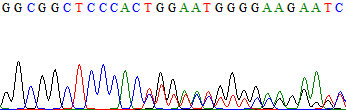


Patient ID: HCM-38

*MYBPC3*: c.2373dupG:p.(Trp792ValfsTer41)


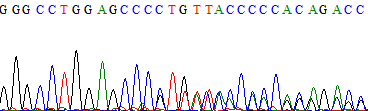


Patient ID: HCM-43

*MYBPC3*: c.2864_2865del:p.(Pro955ArgfsTer95)


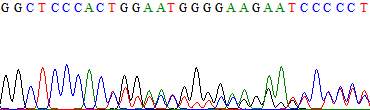


Patient ID: HCM-39

*MYBPC3*: c.2373dupG:p.(Trp792ValfsTer41)


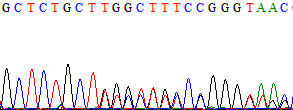


Patient ID: HCM-48

*MYBPC3*: c.3605delG:p.(Cys1202Leufs*35)


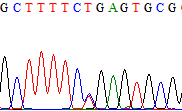


Patient ID: HCM-6

*MYBPC3*: c.2827C>T:p.(Arg943X)


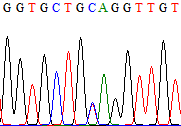


Patient ID: HCM-45

*MYBPC3*: c.3181C>T :p.(Gln1061X)


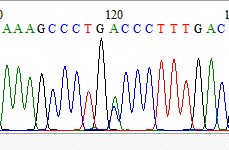


Patient ID: HCM-49

*MYBPC3*:c.3732C>A:p.(Cys1244X)


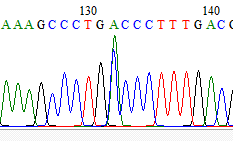


Patient ID: HCM-50

*MYBPC3*: c.3732C>A:p.(Cys1244X)


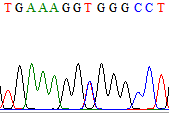


Patient ID: HCM-17

*MYBPC3*: c.1351+2T>C


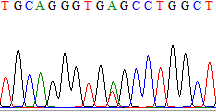


Patient ID: HCM-19

*MYBPC3*: c.1624+4A>T


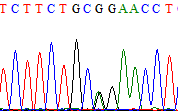


Patient ID: HCM-34

*MYBPC3*: c.1928-2A>G


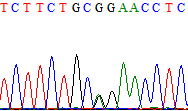


Patient ID: HCM-29

*MYBPC3*: c.1928-2A>G


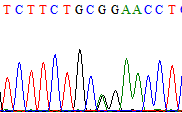


Patient ID: HCM-30

*MYBPC3*: c.1928-2A>G


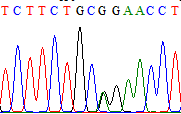


Patient ID: HCM-31

*MYBPC3*: c.1928-2A>G


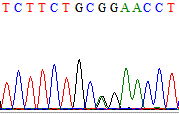


Patient ID: HCM-32

*MYBPC3*: c.1928-2A>G


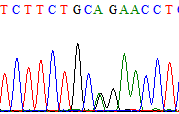


Patient ID: HCM-5

*MYBPC3*: c.1928-2A>G


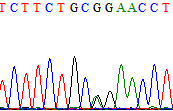


Patient ID: HCM-33

*MYBPC3*: c.1928-2A>G


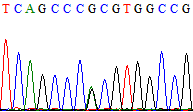


Patient ID: HCM-3

*MYBPC3*: c.530G>A:p.(Arg177His)


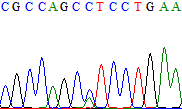


Patient ID: HCM-5

*MYBPC3*: c.547C>A:p.(Leu183Ile)


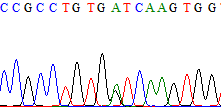


Patient ID: HCM-7

*MYBPC3*: c.565G>A:p.(Val189Ile)


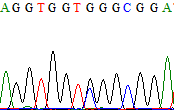


Patient ID: HCM-11

*MYBPC3*: c.841C>G:p.(Arg281Gly)


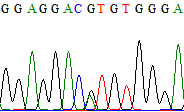


Patient ID: HCM-15

*MYBPC3*: c.961G>A:p.(Val321Met)


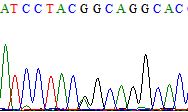


Patient ID: HCM-13

*MYBPC3*: c.977G>A:p.(Arg326Gln)


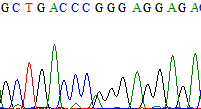


Patient ID: HCM-20

*MYBPC3*: c.1484G>A:p.(Arg495Gln)


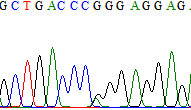


Patient ID: HCM-21

*MYBPC3*: c.1484G>A:p.(Arg495Gln)


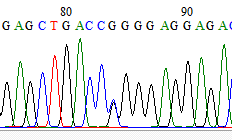


Patient ID: HCM-22

*MYBPC3*:c.1483C>G:p.(Arg495Gly)


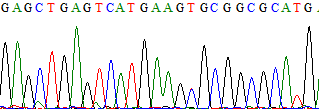


Patient ID: HCM-40

*MYBPC3*: c.2498C>T:p.(Ala833Val)


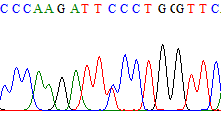


Patient ID: HCM-51

*MYBPC3*: c.3637T>C:p.(Ser1213Pro)


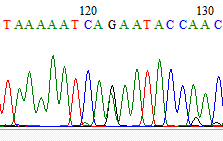


Patient ID: HCM-28

*MYBPC3*: c.1831G>A:p.(Glu611Lys)


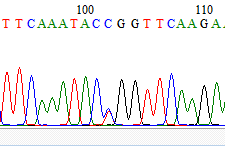


Patient ID: HCM-23

*MYBPC3*: c.1504C>T:p.(Arg502Trp)


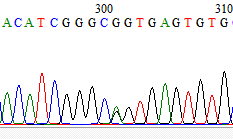


Patient ID: HCM-27

*MYBPC3*: c.1790G>A:p.(Arg597Gln)


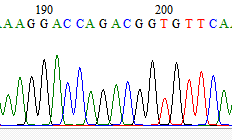


Patient ID: HCM-26

*MYBPC3*: c.1684G>A:p.(Ala562Thr) (HOM)


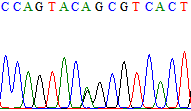


Patient ID: HCM-14

*MYBPC3*: c.1021G>A:p.(Gly341Ser)


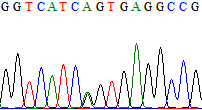


Patient ID: HCM-35

*MYBPC3*: c.2308G>A:p.(Asp770Asn)


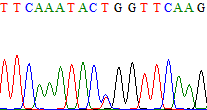


Patient ID: HCM-24

*MYBPC3*: c.1504C>T:p.(Arg502Trp)


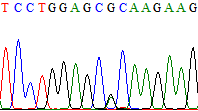


Patient ID: HCM-41

*MYBPC3*: c.2429G>A:p.(Arg810His)


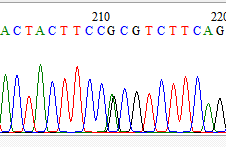


Patient ID: Patient ID: HCM-46

*MYBPC3*: c.3413G>A:p.(Arg1138His)


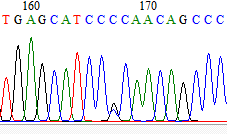


Patient ID: Patient ID: HCM-44

*MYBPC3*: c.3065G>C:p.(Arg1022Pro)


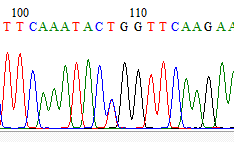


Patient ID: HCM-25

*MYBPC3*: c.1504C>T:p.(Arg502Trp)

***MYH7* variants**


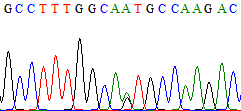


Patient ID: HCM-52

*MYH7*: c.695A>G:p.(Asn232Ser)


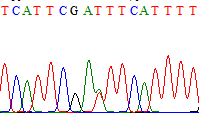


Patient ID: HCM-53

*MYH7*: c.748A>T:p.(Ile250Phe)


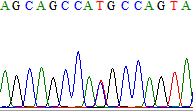


Patient ID: HCM-54

*MYH7*: c.1357C>T:p.(Arg453Cys)


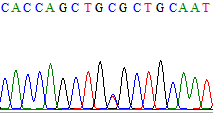


Patient ID: HCM-56

*MYH7*: c.2080C>T:p.(Arg694Cys)


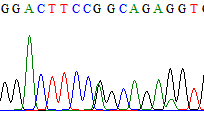


Patient ID: HCM-57

*MYH7*: c.2156G>A:p.(Arg719Gln)


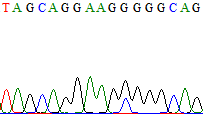


Patient ID: HCM-58

*MYH7*: c.2221G>C:p.(Gly741Arg)


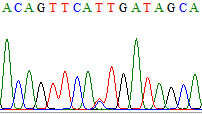


Patient ID: HCM-59

*MYH7*: c.2207T>C:p.(Ile736Thr)


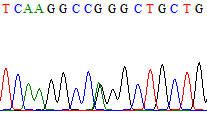


Patient ID: ID=19774-MP-01-065

*MYH7*: c.2302G>A:p.(Gly768Arg)


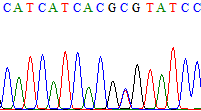


Patient ID: HCM-61

*MYH7*: c.2359C>T:p.(Arg787Cys)


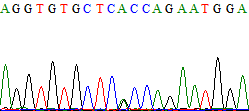


Patient ID: HCM-60

*MYH7*: c.2389G>A:p.(Ala797Thr)


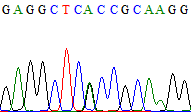


Patient ID: HCM-63

*MYH7*: c.2606G>A:p.(Arg869His)


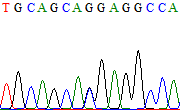


Patient ID: HCM-44

*MYH7*: c.3555C>G:p.(His1185Gln)


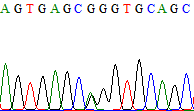


Patient ID: HCM-65

*MYH7*: c.5135G>A:p.(Arg1712Gln)


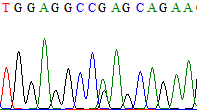


Patient ID: HCM-66

*MYH7*: c.5485G>A:p.(Glu1829Lys)

***MYH6* variants**


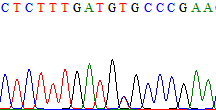


Patient ID: HCM-68

*MYH6*: c.2579G>A:p.(Arg860His)


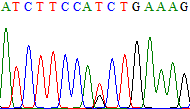


Patient ID: HCM-67

*MYH6*: c.831G>T:p.(Gln277His)


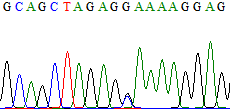


Patient ID: HCM-71

*MYH6*: c.3883G>C:p.(Glu1295Gln)


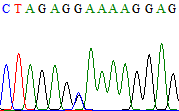


Patient ID: HCM-72

*MYH6*: c.3883G>C:p.(Glu1295Gln)


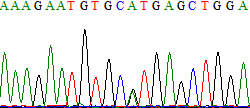


Patient ID: HCM-52

*MYH6*: c.4577A>G:p.(His1526Arg)


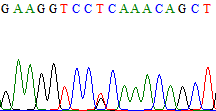


Patient ID: HCM-40

*MYH6*: c.4595G>T:p.(Arg1532Leu)

***MYL2* variant**


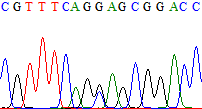


Patient ID: HCM-80

*MYL2*: c.275G>C:p.(Gly92Ala)

***MYL3* variants**


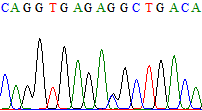


Patient ID: HCM-79

*MYL3*: c.488G>C:p.(Arg163Thr)


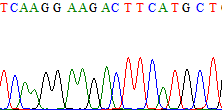


Patient ID: HCM-77

*MYL3*: c.170C>A:p.(Ala57Asp) (HOM)


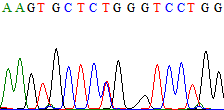


Patient ID: HCM-78

*MYL3*: c.280C>T:p.(Arg94Cys)

***MYLK2* variants**


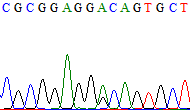


Patient ID: HCM-76

*MYLk2*: c.266G>A:p.(Gly89Asp)


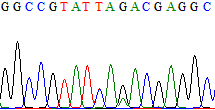


Patient ID: HCM-63

*MYLk2*: c.1529C>T:p.(Ser510Leu)

***TNNT2* variants**


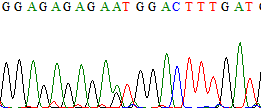


Patient ID: HCM-73

*TNNT2*: c.283G>A:p.(Val95Met)


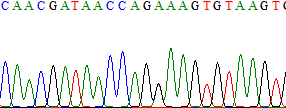


Patient ID: HCM-74

*TNNT2*: c.847A>G:p.(Lys283Glu)

***FLNC* variants**


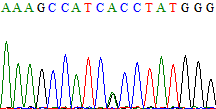


Patient ID: HCM-92

*FLNC*: c.838G>A:p.(Ala280Thr)

Patient ID: HCM-93

*FLNC*: c.2078A>C:p.(Asp693Ala)


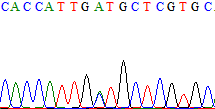


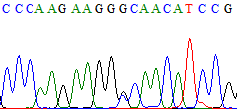


Patient ID: HCM-94

*FLNC*: c.4763C>G:p.(Ala1588Gly)


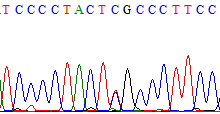


Patient ID: HCM-95

*FLNC*: c.4871C>T:p.(Ser1624Leu)


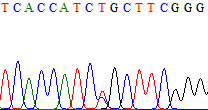


Patient ID: HCM-66

*FLNC*: c.5155C>T:p.(Arg1719Cys)


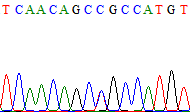


Patient ID: HCM-96

*FLNC*: c.5578C>T:p.(Arg1860Cys)


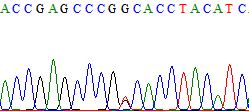


Patient ID: HCM-97

*FLNC*: c.6317G>T:p.(Gly2106Val)


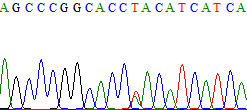


Patient ID: HCM-98

*FLNC*: c.6322T>A:p.(Tyr2108Asn)


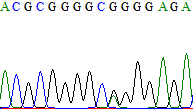


Patient ID: HCM-99

*FLNC*: c.6595G>A:p.(Gly2199Arg)


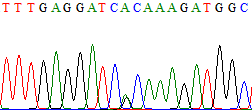


Patient ID: HCM-100

*FLNC*: c.7091G>A:p.(Arg2364His)


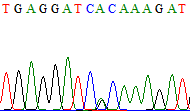


Patient ID: HCM-101

*FLNC*: c.7091G>A:p.(Arg2364His)


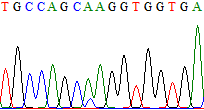


Patient ID: HCM-102

*FLNC*: c.7909A>C:p.(Lys2637Gln)

***ALPK3* variants**


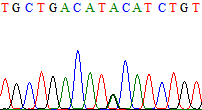


Patient ID: HCM-86

*ALPK3*: c.1490A>G:p.(Tyr497Cys)


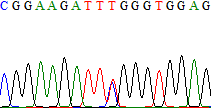


Patient ID: HCM-87

*ALPK3*: c.4447C>T:p.(Arg1483Trp)


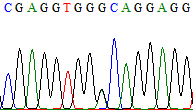


Patient ID: HCM-88

*ALPK3*: c.4565G>A:p.(Gly1522Asp)


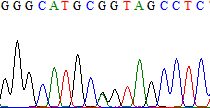


Patient ID: HCM-76

*ALPK3*: c.5720G>A:p.(Arg1907Gln)

***FHOD3* variants**


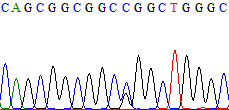


Patient ID: HCM-103

*FHOD3*: c.80G>C:p.(Arg27Pro)


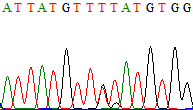


Patient ID: HCM-104

*FHOD3*: c.531G>T:p.(Leu177Phe)


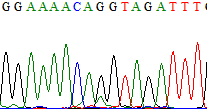


Patient ID: HCM-105

*FHOD3*: c.1646G>A:p.(Ser549Asn)


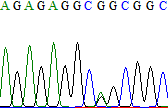


Patient ID: HCM-106

*FHOD3*: c.1910G>A:p.(Arg637Gln)


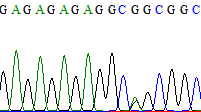


Patient ID: HCM-48

*FHOD3*: c.1910G>A:p.(Arg637Gln)


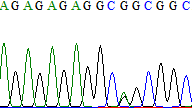


Patient ID: HCM-27

*FHOD3*: c.1910G>A:p.(Arg637Gln)


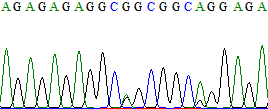


Patient ID: HCM-107

*FHOD3*: c.1910G>A:p.(Arg637Gln)


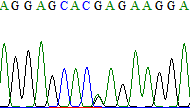


Patient ID: HCM-109

*FHOD3*: c.2059G>A:p.(Glu687Lys)


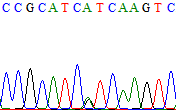


Patient ID: HCM-110

*FHOD3*: c.4708G>A:p.(Val1570Ile)

***ACTN2* variants**


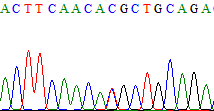


Patient ID: HCM-9

*ACTN2*: c.1040C>T:p.(Thr347Met)


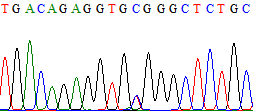


Patient ID: HCM-121

*ACTN2*: c.1312C>T:p.(Arg438Trp)


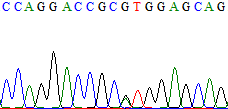


Patient ID: HCM-116

*ACTN2*: c.1372G>A:p.(Val458Met)


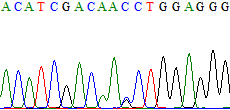


Patient ID: HCM-122

*ACTN2*: c.2082G>C:p.(Lys694Asn)

Patient ID: HCM-123

*ACTN2*: c.2552G>A:p.(Arg851His)

***CSRP3* variants**

Patient ID: HCM-81

*CSRP3*: c.10T>C:p.(Trp4Arg)

Patient ID: HCM-82

*CSRP3*: c.10T>C:p.(Trp4Arg)

Patient ID: HCM-83

*CSRP3*: c.88T>C:p.(Phe30Leu)

***NEXN* variants**

Patient ID: HCM-89

*NEXN*: c.995A>C:p.(Glu332Ala)

Patient ID: HCM-90

*NEXN*: c.995A>C:p.(Glu332Ala)

***CAV3* variants**

Patient ID: HCM-52

*CAV3*: c.233C>T:p.(Thr78Met)

Patient ID: HCM-91

*CAV3*: c.233C>T:p.(Thr78Met)

***MYPN* variants**

Patient ID: HCM-124

*MYPN*: c.578A>G:p.(Gln193Arg)

Patient ID: HCM-100

*MYPN*: c.3335C>T:p.(Pro1112Leu)

Patient ID: HCM-125

*MYPN*: c.3335C>T:p.(Pro1112Leu)

Patient ID: HCM-126

*MYPN*: c.3335C>T:p.(Pro1112Leu)

Patient ID: HCM-127

*MYPN*: c.3421G>A:p.(Ala1141Thr)

***TCAP*  variants**

Patient ID: HCM-85

*TCAP*: c.212T>C:p.(Met71Thr)

***VCL* variants**

Patient ID: HCM-91

*VCL*: c.853C>T:p.(Arg285Cys)
