# Supplementary material for: Exome sequencing data reanalysis of 200 hypertrophic cardiomyopathy patients: the HYPERGEN French cohort 5 years after the initial analysis
Source: Front Med (Lausanne). 2024 Oct 31;11:1480947. doi: 10.3389/fmed.2024.1480947 (PMC11565434; doi:10.3389/fmed.2024.1480947)
Supplement: Supplementary file 3 [file Table_3.DOCX]

| **Gene Symbol** | **Gene Name** | ***OMIM** | **MIM (HCM) Phenotype** | **HCM**  **MIM number** | **Inheritance** | **Other phenotypes** | **Phenotype**  **MIM number** | **Inheritance** |
| --- | --- | --- | --- | --- | --- | --- | --- | --- |
| *MYBPC3* | myosin binding protein C3 | * 600958 | Cardiomyopathy, familial hypertrophic, 4 | 115197 | AD,AR | Cardiomyopathy, dilated, 1MM | 615396 | AD |
|  |  |  |  |  |  | Left ventricular noncompaction 10 | 615396 |  |
| *MYH7* | myosin heavy chain 7 | * 160760 | Cardiomyopathy, familial hypertrophic, 1 | 192600 | AD,DD | Cardiomyopathy, dilated, 1S | 613426 | AD,AR |
|  |  |  |  |  |  | Congenital myopathy 7A, myosin storage | 608358 |  |
|  |  |  |  |  |  | Congenital myopathy 7B, myosin storage | 255160 |  |
|  |  |  |  |  |  | Laing distal myopathy | 160500 |  |
|  |  |  |  |  |  | Left ventricular noncompaction 5 | 613426 |  |
| *MYL2* | myosin light chain 2 | * 160781 | Cardiomyopathy, familial hypertrophic, 10 | 608758 | AD | Myopathy, myofibrillar, 12, infantile-onset, with cardiomyopathy | 619424 | AR |
| *MYL3* | myosin light chain 3 | * 160790 | Cardiomyopathy, familial hypertrophic, 8 | 608751 | AD,AR | NA | NA | NA |
| *TNNT2* | troponin T2, cardiac type | * 191045 | Cardiomyopathy, familial hypertrophic, 2 | 115195 | AD | Cardiomyopathy, dilated, 1D | 601494 | AD |
|  |  |  |  |  |  | Cardiomyopathy, familial restrictive, 3 | 612422 |  |
|  |  |  |  |  |  | Left ventricular noncompaction 6 | 601494 |  |
| *MYLK2* | myosin light chain kinase 2 | * 606566 | Cardiomyopathy, hypertrophic, 1, digenic | 192600 | AD,DD | NA | NA | NA |
| *CSRP3* | cysteine and glycine rich protein 3 | * 600824 | Cardiomyopathy, hypertrophic, 12 | 612124 | AD | ?Cardiomyopathy, dilated, 1M | 607482 | NA |
| *MYH6* | myosin heavy chain 6 | * 160710 | Cardiomyopathy, familial hypertrophic, 14 | 613251 | AD | Atrial septal defect 3 | 614089 | AD |
|  |  |  |  |  |  | Cardiomyopathy, dilated, 1EE | 613252 |  |
|  |  |  |  |  |  | {Sick sinus syndrome 3} | 614090 |  |
| *ACTN2* | actinin alpha 2 | * 102573 | Cardiomyopathy, hypertrophic, 23, with or without LVNC | 612158 | AD | Cardiomyopathy, dilated, 1AA, with or without LVNC | 612158 | AD |
|  |  |  |  |  |  | Congenital myopathy 8 | 618654 |  |
|  |  |  |  |  |  | Myopathy, distal, 6, adult onset | 618655 |  |
| *ALPK3* | alpha kinase 3 | * 617608 | Cardiomyopathy, familial hypertrophic 27 | 618052 | AR | NA | NA | NA |
| *VCL* | vinculin | * 193065 | Cardiomyopathy, hypertrophic, 15 | 613255 | AD | Cardiomyopathy, dilated, 1W | 611407 | NA |
| *JPH2* | junctophilin 2 | * 605267 | Cardiomyopathy, hypertrophic, 17 | 613873 | AD | Cardiomyopathy, dilated, 2E | 619492 | AR |
| *NEXN* | nexilin F-actin binding protein | * 613121 | Cardiomyopathy, hypertrophic, 20 | 613876 | AD | Cardiomyopathy, dilated, 1CC | 613122 | AD |
| *MYPN* | myopalladin | * 608517 | Cardiomyopathy, hypertrophic, 22 | 615248 | AD | Cardiomyopathy, dilated, 1KK | 615248 | AD, AR |
|  |  |  |  |  |  | Cardiomyopathy, familial restrictive, 4 | 615248 |  |
|  |  |  |  |  |  | Nemaline myopathy 11 | 617336 |  |
| *TCAP* | titin-cap | * 604488 | Cardiomyopathy, hypertrophic, 25 | 607487 | AD | Muscular dystrophy, limb-girdle,7 | 601954 | AR |
| *FLNC* | filamin C | * 102565 | Cardiomyopathy, familial hypertrophic, 26 | 617047 | AD | Cardiomyopathy, familial restrictive 5 | 617047 | AD |
|  |  |  |  |  |  | Myopathy, distal, 4 | 614065 |  |
|  |  |  |  |  |  | Myopathy, myofibrillar, 5 | 609524 |  |
| *FHOD3* | formin homology 2 domain containing 3 | * 609691 | Cardiomyopathy, familial hypertrophic, 28 | 619402 | AD | NA | NA | NA |
| *CAV3* | caveolin 3 | * 601253 | Cardiomyopathy, familial hypertrophic | 192600 | AD, DD | Creatine phosphokinase, elevated serum | 123320 | AD |
|  |  |  |  |  |  | Long QT syndrome 9 | 611818 |  |
|  |  |  |  |  |  | Myopathy, distal, Tateyama type | 614321 |  |
|  |  |  |  |  |  | Rippling muscle disease 2 | 606072 |  |
| *TRIM63* | tripartite motif containing 63 | * 606131 | NA | NA | AD,AR | NA | NA | NA |
| *SVIL* | supervillin | * 604126 | NA | NA | NA | Myofibrillar myopathy 10 | 619040 | AR |

AD: Autosomal dominant; AR: Autosomal recessive; DD: Digenic dominant
